# K^+^ Channel-SEC11 Binding Exchange Regulates SNARE Assembly for Secretory Traffic^1^

**DOI:** 10.1104/pp.19.00919

**Published:** 2019-09-23

**Authors:** Sakharam Waghmare, Cecile Lefoulon, Ben Zhang, Edita Liliekyte, Naomi Donald, Michael R. Blatt

**Affiliations:** Laboratory of Plant Physiology and Biophysics, Bower Building, University of Glasgow, Glasgow G12 8QQ, United Kingdom

## Abstract

Cell expansion requires that ion transport and secretory membrane traffic operate in concert. Evidence from Arabidopsis (*Arabidopsis thaliana*) indicates that such coordination is mediated by physical interactions between subsets of so-called SNARE (soluble N-ethylmaleimide-sensitive factor attachment protein receptor) proteins, which drive the final stages of vesicle fusion, and K^+^ channels, which facilitate uptake of the cation to maintain cell turgor pressure as the cell expands. However, the sequence of SNARE binding with the K^+^ channels and its interweaving within the events of SNARE complex assembly for exocytosis remains unclear. We have combined protein-protein interaction and electrophysiological analyses to resolve the binding interactions of the hetero-oligomeric associations. We find that the RYxxWE motif, located within the voltage sensor of the K^+^ channels, is a nexus for multiple SNARE interactions. Of these, K^+^ channel binding and its displacement of the regulatory protein SEC11 is critical to prime the Qa-SNARE SYP121. Our results indicate a stabilizing role for the Qbc-SNARE SNAP33 in the Qa-SNARE transition to SNARE complex assembly with the R-SNARE VAMP721. They also suggest that, on its own, the R-SNARE enters an anomalous binding mode with the channels, possibly as a fail-safe measure to ensure a correct binding sequence. Thus, we suggest that SYP121 binding to the K^+^ channels serves the role of a primary trigger to initiate assembly of the secretory machinery for exocytosis.

## Abstract (precis)

K^+^ channels initiate a binding exchange with the SNARE SYP121 to trigger SNARE complex assembly and drive secretory vesicle fusion at the plant plasma membrane.

SNARE (soluble N-ethylmaleimide-sensitive factor attachment protein receptor) proteins have important roles in membrane traffic. In plants, they contribute to cell homeostasis and responses to biotic and abiotic stress, and they are vital for development and morphogenesis (Shope and Mott, 2006; Campanoni and Blatt, 2007; Kwon et al., 2008; Eisenach et al., 2012). SNAREs facilitate the final steps of vesicle fusion, assembling in complex to bring membrane surfaces together and drive intercalation of the vesicle and target membrane bilayers. SNAREs have been classified as target and vesicle SNAREs, depending on their functional localization, and as Q (Gln)- and R (Arg)-SNAREs according to the conserved residues contributing to the central layer formed in complex (Fasshauer et al., 1998b). The assembled SNARE complex brings together the SNARE subunits, each containing the Qa-, Qb-, Qc-, or R-SNARE motif (Jahn and Scheller, 2006; Karnik et al., 2017).

It is widely accepted that SNARE complex assembly begins with the Qa-SNARE transition from the “closed” to the “open” conformation (Pratelli et al., 2004; Jahn and Scheller, 2006; Brunger et al., 2009; Rizo and Südhof, 2012). In the closed state, the N-terminal Habc α-helices fold back onto the central H3 domain of the Qa-SNARE to prevent interaction with its cognate SNARE partners and thereby regulate Qa-SNARE availability for complex assembly (Fasshauer et al., 1998a; Weber et al., 1998; Jahn and Scheller, 2006). The closed conformation of many Qa-SNAREs is stabilized by members of the SEC1/MUNC18 (SM) family of proteins and its release depends on additional protein interactions. In neurons, binding of the ancillary protein MUNC13 triggers conformational changes in MUNC18 that facilitate Qa-SNARE transit to the open conformation and expose the H3 domain. MUNC18 also binds and stabilizes the assembled SNARE core complex, thereby promoting vesicle fusion. Thus, for neuronal secretion, the binding between the SM proteins MUNC18 and MUNC13 initiates an important transition regulating Qa-SNARE availability that leads to SNARE complex assembly (Burgoyne and Morgan, 2007; Ma et al., 2013; Demircioglu et al., 2014).

In Arabidopsis (*Arabidopsis thaliana*), most secretory traffic at the plasma membrane is driven by the Qa-SNAREs SYR1/PEN1 (SYP121) and SYP122 through their assembly with the near-identical cognate R-SNAREs VAMP721 and VAMP722 and the Qbc-SNARE SNAP33 (Sanderfoot, 2007; Bassham and Blatt, 2008; Kwon et al., 2008; Enami et al., 2009; Karnik et al., 2013b, 2015; Fujiwara et al., 2014; Zhang et al., 2015). The Arabidopsis SM protein SEC11 associates preferentially with SYP121 and, like its mammalian counterparts, will stabilize both the closed Qa-SNARE and the core complex it assembles (Karnik et al., 2013b, 2015). Arabidopsis also expresses a putative MUNC13 ortholog, PATROL1, that is important for H^+^-ATPase traffic to the plasma membrane; however, PATROL1 has no impact on traffic mediated by these Qa-SNAREs (Hashimoto-Sugimoto et al., 2013). Thus, it remains unclear how SEC11 (de)binding with SYP121 is facilitated to promote secretion.

Evidence gathered over the past decade has uncovered several other protein partners of SYP121 that point to an unconventional mechanism for its regulation. Indeed, SYP121 was first identified as a factor essential for regulation by the phytohormone abscisic acid (ABA) of ion channels in stomatal guard cells leading to stomatal closure under water stress (Leyman et al., 1999, 2000; Geelen et al., 2002). We now know that SYP121 has unique roles in responses to drought and ABA (Leyman et al., 1999; Eisenach et al., 2012). Most important, through the F^9^xRF motif near its N terminus, SYP121 binds directly with a subset of K^+^ channels, including KAT1 and KC1, already present at the plasma membrane to promote channel gating (Honsbein et al., 2009, 2011; Grefen et al., 2010). The K^+^ channels incorporate a unique RYxxWE motif at the cytosolic surface of their voltage sensor domain (VSD), and binding of the SNARE through this motif effectively commandeers the voltage-dependent conformations of the VSD to enhance secretion in parallel with voltage-driven K^+^ uptake (Grefen et al., 2015; Lefoulon et al., 2018). Significantly, both binding motifs are unique to land plants and are highly conserved within a subset of plasma membrane K^+^ channels and Qa-SNAREs, suggesting that their functional coordination was an important evolutionary step in the colonization of the dry land environment (Honsbein et al., 2011; Grefen et al., 2015; Karnik et al., 2017).

The cognate R-SNAREs VAMP721 and VAMP722 also bind with these K^+^ channels, but in contrast with SYP121, R-SNARE binding suppresses channel gating (Zhang et al., 2015). The R-SNAREs incorporate, within their regulatory (so-called longin) domain, a linear binding motif for the channels of GHTFNYLVExGxxY centered around Tyr-57 (Zhang et al., 2015, 2017). At present, we do not know the complementary motif on the K^+^ channels. However, since overall the channel-SNARE interactions promote vesicle traffic with K^+^ uptake (Grefen et al., 2015), it is likely that a binding exchange with the K^+^ channels occurs between the SNAREs, possibly during SNARE complex assembly. Intriguingly, the SYP121 F^9^xRF channel-binding motif overlaps with one of the two sites for SEC11 binding at the Qa-SNARE N terminus (Grefen et al., 2010). This SEC11 binding site is highly conserved and is thought to be important for SYP121 transit from the closed to the open conformation (Karnik et al., 2013b, 2015). Thus, a more complex sequence of binding exchanges may be engaged with SEC11, the Qa-SNAREs, and R-SNAREs, and centered on the K^+^ channels.

Here, we have analyzed the coordinate binding interactions of SYP121 and its cognate partners with SEC11 and the K^+^ channels KC1 and KAT1. We show that the cognate SNAREs each recognize a common binding motif on the K^+^ channels that underpins sets of competitive interactions, several with consequences for channel gating. Using gating as a proxy for function, we uncover differential effects of SEC11 binding that are consistent with a subset of binding exchanges essential for SNARE complex assembly. These and additional data lead us to propose that the K^+^ channels fill a functional role analogous to that of MUNC13, but in a manner that engages membrane voltage to trigger the final protein binding events that drive vesicle fusion.

## RESULTS

### VAMP721, SNAP33, and SEC11 bind the K^+^ Channel VSD

We used the mating-based split-ubiquitin system (mbSUS) assay, previously employed successfully to identify K^+^ channel binding motifs (Honsbein et al., 2009; Grefen et al., 2010; Karnik et al., 2015; Zhang et al., 2015, 2017; Horaruang and Zhang, 2017), to assess channel binding with the cognate SNAREs and identify the relevant binding motifs. The mbSUS assay takes advantage of protein fusions with the N- and C-terminal halves (Nub-X and Y-Cub) of ubiquitin. Reassembly of ubiquitin leads to transactivator cleavage from the Y-Cub fusion, its transit to the nucleus, reporter gene activation, and yeast growth on selective media. The assay offers a number of advantages over other yeast-based screens for protein interactions, including bait suppression in the presence of Met as a test for interaction specificity and the possibility of using full-length, integral membrane proteins.

**Figure 1.**
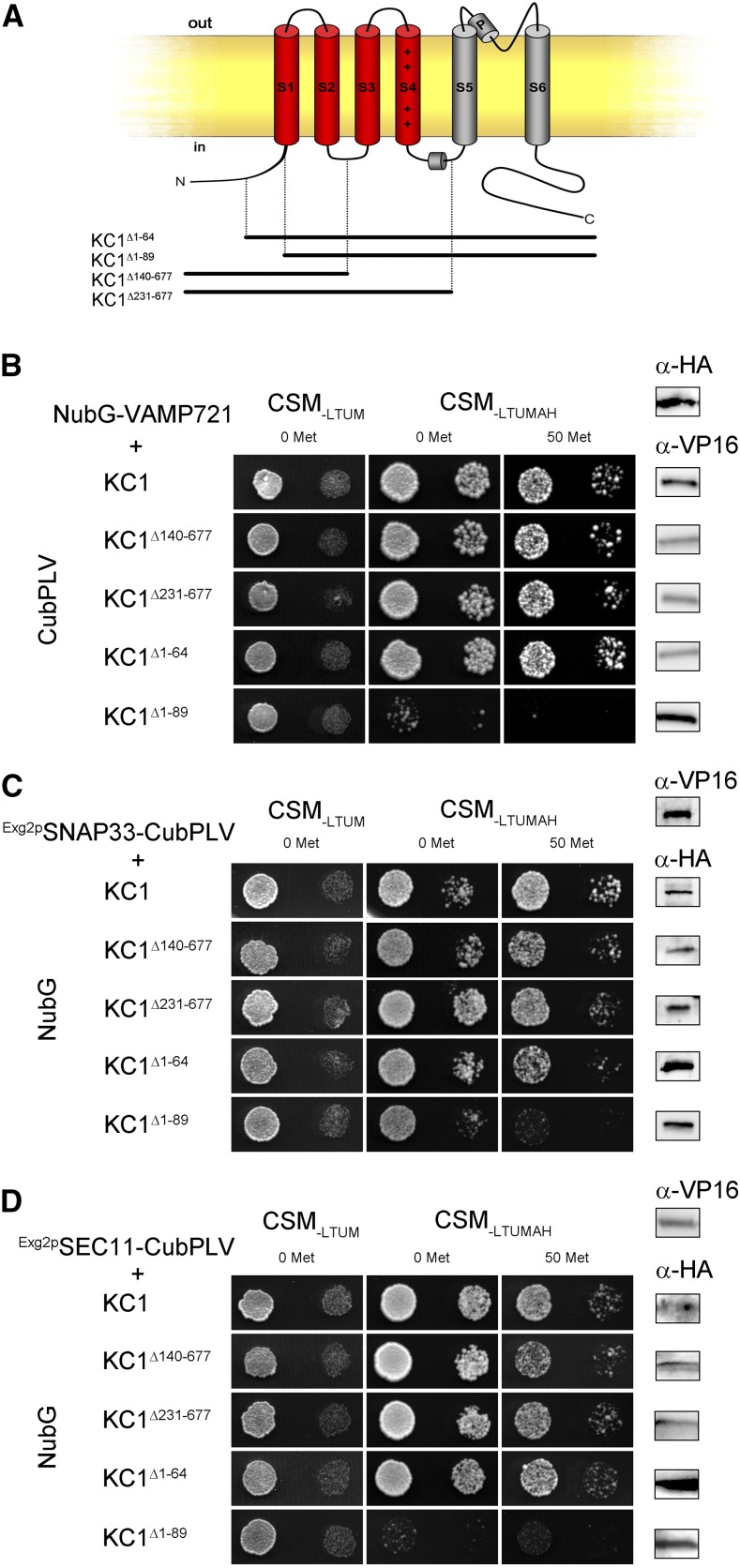
KC1 K^+^ channel interacts with VAMP721, SNAP33, and SEC11 via a cytosolic N-terminal region of its voltage sensor domain. A, Schematic of Kv channel structure with the VSD comprising α-helices S1 to S4 identified in red and the pore-lining α-helices S5 and S6 in gray (adapted from Grefen et al. [2015]). Segments expressed for the KC1 deletions are indicated below. B to d, Yeast mbSUS assay for interaction of the VAMP721, SNAP33, and SEC11 fusions with KC1 and its deletion constructs (A) as bait with Y-Cub fusions. VAMP721, SNAP33, and SEC11 served as NubG-X prey fusions and SNAP33 and SEC11 were anchored via the GPI signal peptide (Zhang et al., 2018). Positive and negative controls are included in Supplemental Figure S1. Similar results were obtained in three independent experiments. Growth on CSM_-LTUM_ was used to verify the presence of both bait and prey expression. CSM_-LTUMAH_ was used to verify Ade- and His-independent growth of the yeast diploids. The addition of 50 μm Met to CSM_-LTUMAH_ suppressed bait expression as a test for interaction specificity. Yeast was dropped at 1.0 and 0.1 OD_600_ in each case. Incubation time was 24 h for the CSM_-LTUM_ plate and 72 h for CSM_-LTUMAH_ plates. Immunoblot analysis (5 μg total protein/lane) of the haploid yeast used in mating (right) used the αHA antibody for the prey fusions and the αVP16 antibody for the bait fusions.

Initially we screened for channel binding with an overlapping series of channel truncations. Growth was recovered with the diploid yeast expressing Nub-VAMP721 with KC1 truncations on selective media, even in the presence of 50 μm Met, provided that residues 65 to 89 were present in each of the Nub-K^+^ channel fusion constructs (Fig. 1A; Supplemental Fig. S1). Only with yeast expressing the KC1^Δ1–89^ truncation was little or no growth observed, indicating that the critical motif for binding was localized near the S1 α-helix of the KC1 channel VSD. Assembly of the SNARE complex with SYP121 and VAMP721 draws on the cognate partner SNAP33 and is subject to SEC11 (de)binding (Karnik et al., 2017). Therefore, we examined channel binding with these proteins. For mbSUS assays with the otherwise soluble baits SNAP33 and SEC11, we incorporated a glycosylphosphatidylinositol (GPI) anchor (Zhang et al., 2018) to express the corresponding protein fusions with NubG-X fusions of the full-length and truncated channel preys. Again, we recovered growth of the diploid yeast with the KC1 truncations, provided that residues 65 to 89 were present in each of the Nub-K^+^ channel fusions (Fig. 1, B and C; Supplemental Fig. S1). Similar results were obtained in three independent experiments.

**Figure 2.**
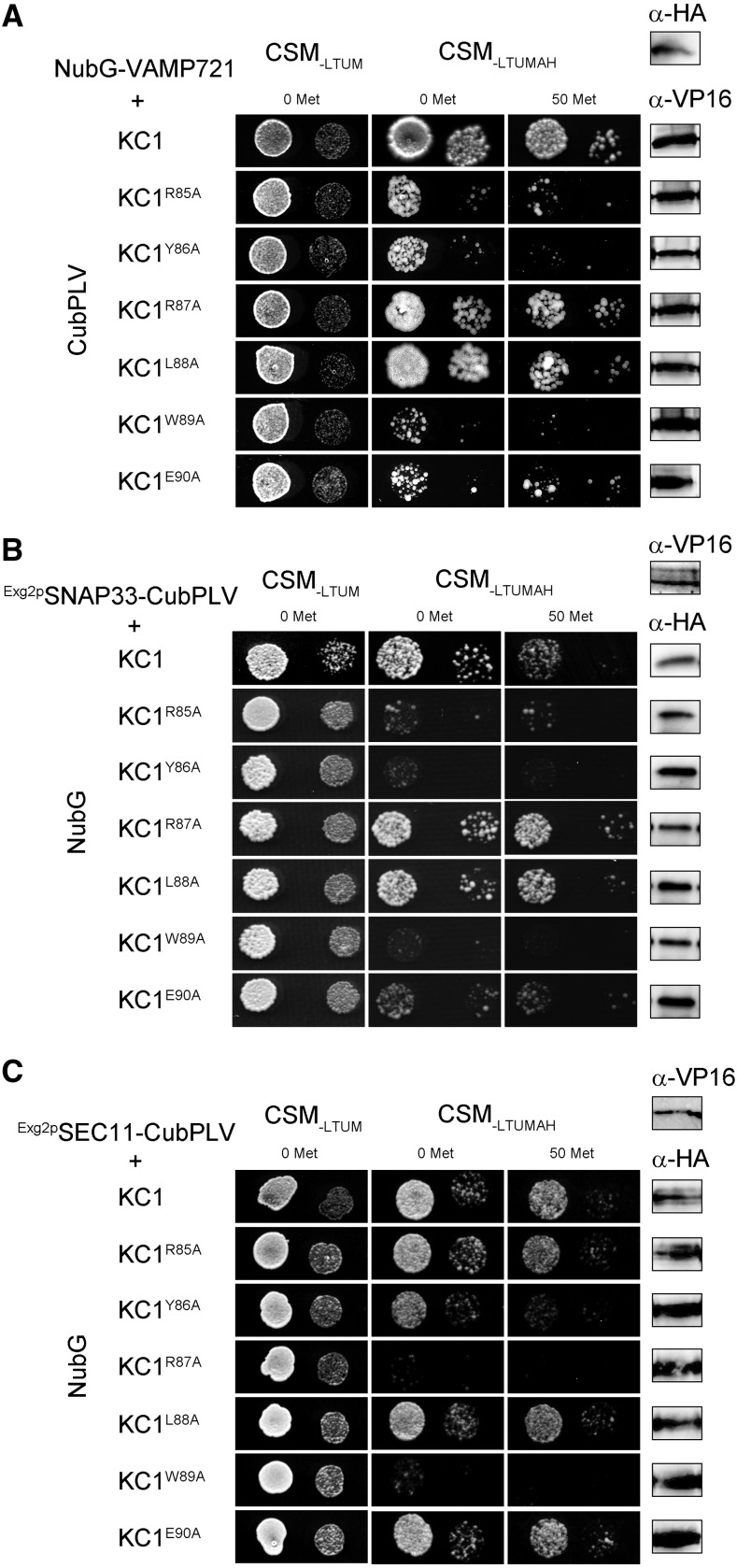
KC1 K^+^ channel interacts with VAMP721, SNAP33, and SEC11 via a common N-terminal motif, RYxxWE, at the base of its VSD. A to C, Yeast mating-based split-ubiquitin assay for interaction of the VAMP721, SNAP33, and SEC11 fusions with KC1 and with Ala substitutions of key residues at the cytosolic face of the VSD as bait with Y–Cub fusions. VAMP721, SNAP33, and SEC11 as NubG-X prey fusions and with SNAP33 and SEC11 anchored via the GPI signal peptide (Zhang et al., 2018). Positive and negative controls are included in Supplemental Figure S1. Similar results were obtained in each of three independent experiments. Growth on CSM_-LTUM_ was used to verify the presence of both bait and prey expression. CSM_-LTUMAH_ was used to verify Ade- and His-independent growth of the yeast diploids. The addition of 50 μm Met to CSM_-LTUMAH_ suppressed bait expression as a test for interaction specificity. Yeast was dropped at 1.0 and 0.1 OD_600_ in each case. Incubation time was 24 h for CSM_-LTUM_ plate and 72 h for CSM_-LTUMAH_ plates. Immunoblot analysis (5 μg total protein/lane) of the haploid yeast used in mating (*right*), used the αHA antibody for the prey fusions and the αVP16 antibody for the bait fusions.

We used Ala scanning mutagenesis in the full-length KC1 channel and mbSUS analysis to isolate the corresponding binding motifs. For VAMP721, the analysis showed that residues R^85^YxxWE at the base of the S1 α-helix were critical for interaction (Fig. 2A; Supplemental Fig. S1). Analysis of KAT1 interactions yielded the same motif, R^58^YxxWE motif in this K^+^ channel (Supplemental Figs. S1 and S2). Similarly, we carried out Ala-scanning mutagenesis in the full-length KC1 channel for motifs essential for SNAP33 and SEC11 binding. In this case, we retransformed yeast with the site-mutated constructs for mbSUS analysis with the GPI-anchored SNAP33 and SEC11 baits (Fig. 2, B and C; Supplemental Figs. S1 and S2). With SNAP33 as the bait, yeast growth was suppressed by Ala substitutions within the same R^85^YxxWE motif of KCl. Parallel analysis with KAT1 indicated a similar dependence on the R^58^YxxWE motif, although some growth was recovered with the KAT1^E63A^ mutant (Supplemental Fig. S2). With SEC11 as the bait, yeast growth was suppressed by Ala substitutions at positions Y^86^RxW of the KC1 channel (Fig. 2C), and a similar pattern, albeit weaker, was observed for KAT1 (Supplemental Fig. S2). Again, similar results were obtained in three independent experiments. These findings indicated that within the base of the S1 α-helix of each K^+^ channel, a linear sequence of residues incorporates overlapping motifs for binding with each of the major components that contribute to SNARE complex assembly with SYP121.

**Figure 3.**
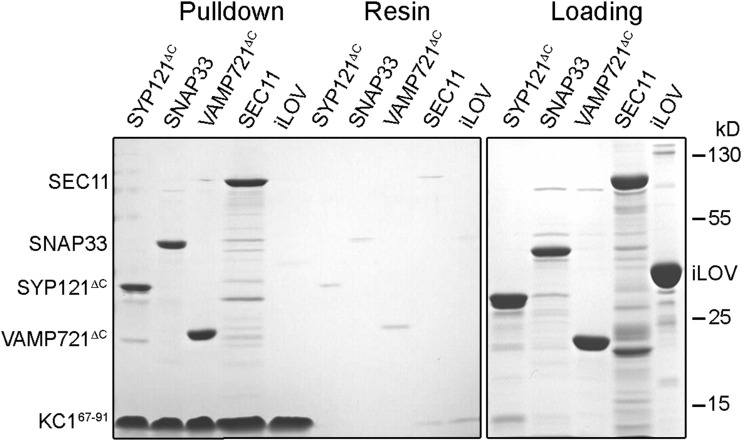
Pulldown analysis of the SNAREs and SEC11 with the KC1^67–91^ cytosolic domain. Tagged proteins were expressed and purified from *Escherichia coli* using His-affinity and size-exclusion chromatography before incubation in vitro overnight at 4°C. Shown is one of three independent pulldown experiments with Coomassie-stained SDS-PAGE analysis following separation with Strep-Tactin resin-immobilized KC1^67–91^-Flag_6_-StrepII. Lane sets are the SDS-PAGE results of (left to right) the pulldown, the resin control, and the loading. Incubations were carried out individually with SYP121^ΔC^-Flag-6His, VAMP721^ΔC^-Flag-6His, Flag_3_-SNAP33-6His, and SEC11-Flag_6_-6His. The unrelated iLOV-6His protein (Christie et al., 1998) was included as a control. Note the lower-molecular weight fractions that are common for purified SEC11 (Karnik et al., 2013b).

To confirm the mbSUS results, we carried out pulldown studies in vitro after expressing and purifying the N-terminal cytosolic domains of the two K^+^ channels. These experiments employed the cytosolic domains of SYP121 and VAMP721 used previously for pulldown studies (Karnik et al., 2013b, 2015; Grefen et al., 2015) and with the soluble, full-length SNAP33 and SEC11 proteins. We expressed and purified the Flag- and His-tagged constructs to carry out pulldown analyses with purified KC1^67–91^-Flag_3_-StrepII and KAT1^1–63^-Flag_6_-StrepII immobilized on a Strep-Tactin affinity resin. After incubation with a 3-fold excess of the prey proteins, unbound protein was washed and protein bound to the resin was eluted and analyzed by SDS-PAGE. We included glutathione *S*-transferase-tagged iLOV protein as a negative control. iLOV is a soluble phototropin from Arabidopsis (Chapman et al., 2008) and is unrelated to either the SNARE or channel proteins. We found (Fig. 3) that the KC1^67–91^ peptide bound individually with each of the interacting partners identified in the mbSUS assays, SYP121^ΔC^, VAMP721^ΔC^, SNAP33, and SEC11, as indicated by the presence of bands of their corresponding sizes and their absence in the resin control. By contrast, no binding to the iLOV protein was recovered. Similar results were obtained with the KAT1^1–63^ bait (Supplemental Fig. S3). Thus, we concluded that the N-terminal cytosolic domain of the K^+^ channels is a prerequisite for binding of all three cognate SNARE partners, as well as the regulatory SM protein SEC11.

### The SNARE Complex Can Incorporate the K^+^ Channel N Terminus

**Figure 4.**
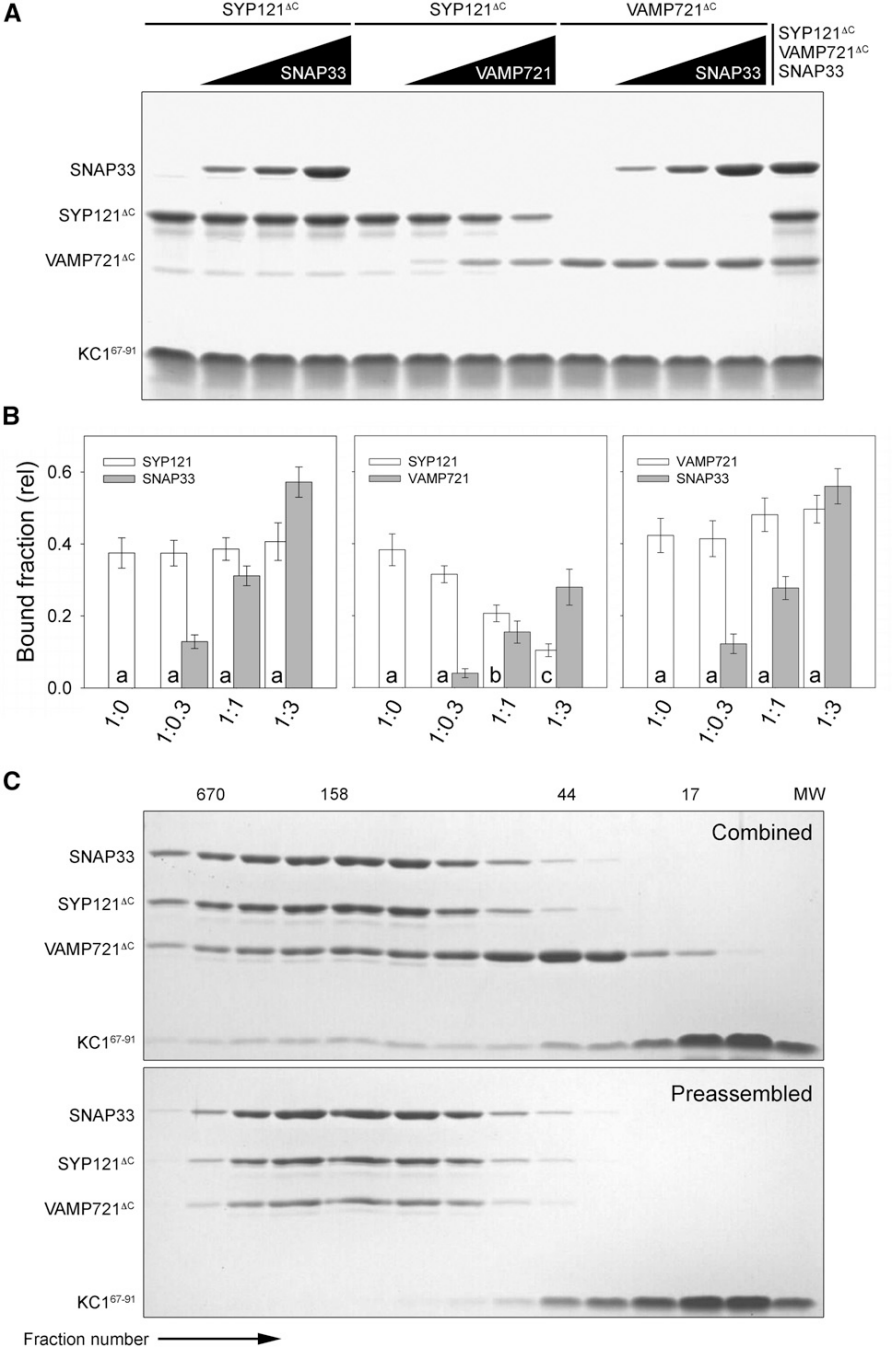
The cytosolic domain KC1^67–91^ associates differentially with the cognate SNAREs in binary combination and in complex. A, Tagged proteins were expressed and purified from *E. coli* using His-affinity and size-exclusion chromatography before incubation in vitro overnight at 4°C. Shown is one of three independent pulldown experiments with Coomassie-stained SDS-PAGE analysis following separation with Strep-Tactin resin-immobilized KC1^67–91^-Flag_3_-StrepII. Lane sets are the SDS-PAGE results of pulldowns with a 3-fold excess of SYP121^ΔC^ and VAMP721^ΔC^ with competing additions of 0, 0.3-, 1-, and 3-fold excess of VAMP721^ΔC^ SNAP33, as indicated (above). Also shown are the pulldown results on incubation of all three SNAREs with the K^+^ channel peptide (right). SNAREs were incubated at 4°C overnight with Strep-Tactin-immobilized K^+^ channel peptide-Flag_3_-StrepII. Fixed concentrations (5 μm) of SYP121^ΔC^ and VAMP721^ΔC^ were used in each case. Similar results for KAT1^1–63^ are shown in Supplemental Figure S4. B, Bound fraction analysis of KC1^61–97^ and KAT1^1–63^ pulldowns from all six independent experiments. Data are means ± se of the ratios of SNAREs to K^+^ channel peptide. Lowercase letters indicate statistical differences at *P* < 0.05. C, Comigration of KC1^67–91^ peptide-SNARE complex on gel filtration. The KC1^67–91^ peptide-SNARE complex assembly reactions were started either by comixing the channel peptide with SYP121^ΔC^, SNAP33, and VAMP721^ΔC^ (above) or by adding the channel peptide following incubation of SYP121^ΔC^, SNAP33, and VAMP721^ΔC^ to preform the SNARE complex. The SNARE complex was formed by incubation overnight at 4°C and purified by gel filtration chromatography, and the homo-oligomeric peak of SNARE complex was then mixed with KC1^67–91^ peptide. Shown are the SDS-PAGE analyses of gel filtration fractions with the SNAREs and channel peptides, with molecular weights (MW) of standards indicated above each column. Note that the channel peptide remained associated with the SNARE complex when comixed with the SNAREs (Combined) but not with the preformed SNARE complex (Preassembled).

The ability of the K^+^ channels to interact via overlapping motifs with all three cognate SNAREs raises the possibility that the SNAREs compete for channel binding. To address this question, we carried out pulldown experiments using the Strep-Tactin-immobilized channel peptides in binary combinations with each of the cognate SNAREs while varying the concentration of either SNAP33 or VAMP721^ΔC^. The results from one experiment with the KC1^67–91^ peptide are shown in Figure 4A. Similar results were obtained in three independent experiments and with the KAT1^1–63^ peptide (Supplemental Fig. S4), and these are summarized together in Figure 4B. We observed no significant change in the band intensity of SYP121^ΔC^ or VAMP721^ΔC^ with increasing SNAP33, suggesting that SNAP33 does not compete for binding with the channel. By contrast, the binary combination of SYP121^ΔC^ and VAMP721^ΔC^ with either KC1^67–91^ or KAT1^1–63^ showed a near-linear exchange in SYP121^ΔC^ binding with that of VAMP721^ΔC^, indicating a competition between the Qa- and R-SNAREs for channel binding. Indeed, we were not able to recover SYP121^ΔC^ and VAMP721^ΔC^ in binary complex alone, suggesting that the Qa- and R-SNAREs, either alone or with the channel N termini, fail to assemble together by contrast with pairings incorporating SNAP33.

To assess whether all three SNAREs might assemble in complex with the channels, we also performed pulldowns in parallel with these experiments by mixing Strep-Tactin-immobilized KC1^67–91^ together with SYP121^ΔC^, SNAP33, and VAMP721^ΔC^. We recovered all three SNAREs (Fig. 4A). Again, similar results were obtained with KAT1^1–63^ (Supplemental Fig. S4). To confirm whether the channel peptide was associated with the fully assembled SNARE complex, we added the KC1^67–91^ peptide together with the SNAREs at the start of incubation as well as to the preincubated SNAREs. In this case, the mixes were separated by size exclusion chromatography. We recovered SYP121^ΔC^, SNAP33, and VAMP721^ΔC^ with the channel peptide when KC1^67–91^ was combined in the mix concurrently with the SNAREs, but when the SNAREs were preassembled in advance to form a SNARE complex, the SNAREs were recovered without inclusion of the KC1^67–91^ peptide (Fig. 4C). A simple interpretation of these results suggests that the SNAREs assemble in complex with the channel N termini when present, but it does not speak to the possible role or mechanics of K^+^ channel binding.

### SYP121 Coordinates with SNAP33 and VAMP721 in K^+^ Channel Gating

Voltage-gated K^+^ (Kv) channel monomers, including those of plant K^+^ channels, assemble as tetramers to form a functional channel pore (Véry and Sentenac, 2003; Tombola et al., 2006; Dreyer and Blatt, 2009). KAT1 assembles as a homotetramer, but its close homolog, the so-called “silent” KC1 channel monomer, normally functions in vivo as a heterotetramer with the AKT1 K^+^ channel subunit (Duby et al., 2008; Honsbein et al., 2009). Both channel assemblies show gating that is significantly altered, with opposing effects, by SYP121 and VAMP721 (Honsbein et al., 2009; Grefen et al., 2010, 2015; Zhang et al., 2015, 2017; Lefoulon et al., 2018). However, it is not known whether these characteristics are retained in combination with the cognate Qbc-SNARE SNAP33. To address this question, we assayed channel gating as a proxy to assess the effects of SNARE protein combinations. KAT1 was expressed alone and in combinations with SYP121, VAMP721, and SNAP33 in *Xenopus laevis* oocytes for analysis by two-electrode voltage clamp (Honsbein et al., 2009; Lefoulon et al., 2018). Using KAT1 avoided the technical complications of coexpressing KC1 with AKT1, CIPK23, and CBL1, which are necessary for KC1-AKT1 channel activity (Xu et al., 2006; Honsbein et al., 2009).

**Figure 5.**
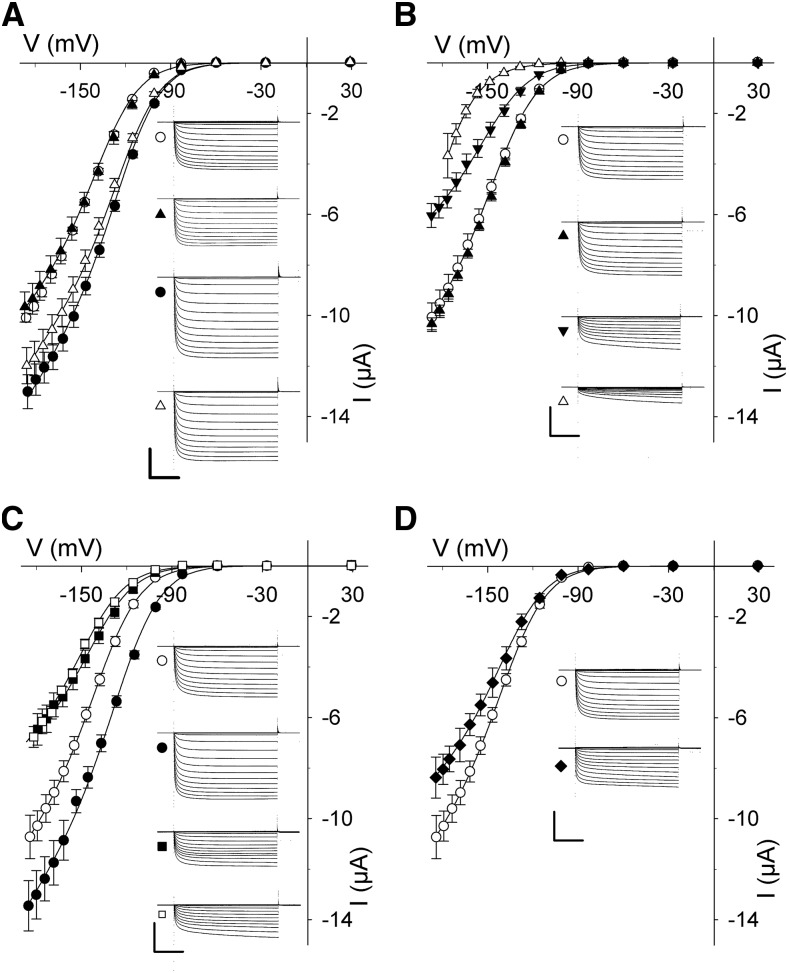
SNAP33 coordinates with its cognate SNAREs in regulating K^+^ channel activity. Mean steady-state voltage curves recorded under voltage clamp from oocytes coexpressing the KAT1 channel alone and with combinations of the cognate SNAREs SYP121, VAMP721, and SNAP33. Data in every case are measurements from at least five independent experiments and are fitted jointly by the nonlinear least-squares method to the Boltzmann function of Equation 1. Representative current traces cross-referenced by symbol are included for each set of curves, as are data for KAT1 alone, for reference. Representative immunoblot analyses and fitting results are included in Supplemental Figure S5. Water-injected controls yielded currents of <300 nA at all voltages and are omitted for clarity. Scales represent 1 s (horizontal), 6 μA (vertical). A, KAT1 alone (○), and coexpressed with SNAP33 (▲), SYP121 (●), and SNAP33 and SYP121 (△). B, KAT1 alone (○), and coexpressed with SNAP33 (▲), VAMP721 (▼), and SNAP33 and VAMP721 (△). C, KAT1 alone (○), and coexpressed with SYP121 (●), VAMP721 (▪), and SYP121 and VAMP721 (□). D, KAT1 alone (○), and coexpressed with SNAP33, VAMP721, and SYP121 (♦).

Figure 5 shows the mean, steady-state current-voltage curves, and representative current traces from at least five independent experiments with oocytes expressing each combination of proteins. To extract the gating characteristics for the K^+^ channel in each case, we fitted the steady-state currents to a Boltzmann function of the form
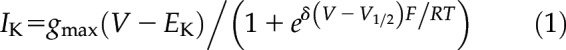


where *g*_max_ is the conductance maximum, *E*_K_ is the K^+^ equilibrium voltage, *V* is the membrane voltage, and *R* and *T* have their usual meanings. Here, the gating characteristic is defined by *V*_1/2_, the midpoint voltage at which the current reaches half-maximal conductance, and by δ, the apparent gating charge that reflects the sensitivity to a change in voltage. In each case, expression of the proteins was verified by immunoblot analysis (Supplemental Fig. S5). When expressed on its own, KAT1 yielded a large, inward-directed current that activated strongly at voltages negative of −100 mV, with a *V*_1/2_ near −135 mV and δ of 1.9 (Supplemental Fig. S5), much as has been reported in the past (Hoshi, 1995; Gajdanowicz et al., 2009; Lefoulon et al., 2018). As before (Grefen et al., 2015; Zhang et al., 2015, 2017; Lefoulon et al., 2018), co-expression with SYP121 and with VAMP721 (Fig. 5, A and B) showed opposing effects on KAT1 gating: SYP121 co-expression displaced *V*_1/2_ to more positive voltages and enhancing *g*_max_, while VAMP721 co-expression displaced *V*_1/2_ to more negative voltages and reduced *g*_max_ when compared with oocytes expressing KAT1 alone. In oocytes coexpressing KAT1 with SNAP33 (Fig. 5A), we observed no appreciable change in KAT1 current and values for *V*_1/2_ and δ were similar to those resolved from measurements from oocytes expressing KAT1 alone. Thus, SNAP33 appeared to have no significant effect on channel activity, even though the protein bound the KAT1 VSD.

We next asked whether SNAP33 might act together with either of its cognate SNAREs to affect KAT1 activity. Again, we recorded K^+^ current under voltage clamp after expressing KAT1 alone, together with binary combinations of the SNAREs SYP121, VAMP721, and SNAP33, and with the tertiary combination of SYP121, SNAP33, and VAMP721, in each case verifying protein expression by immunoblot analysis (Supplemental Fig. S5). Analysis of the results from five independent experiments with each construct combination (Fig. 5, B–D; Supplemental Fig. S5) showed that coexpression of SNAP33 with SYP121 yielded a mean *V*_1/2_ similar to that observed on expressing KAT1 with SYP121 on its own. A substantial impact of SNAP33 on KAT1 gating was observed only in binary combination with VAMP721, which displaced *V*_1/2_ by −25 mV compared to KAT1 alone and by −6 mV compared to KAT1 with VAMP721 (Fig. 5B and Supplemental Fig. S5). We also examined the effect of coexpressing SYP121 with VAMP721; in this case, the result was a displacement of *V*_1/2_ similar to that with VAMP721 alone, suggesting a dominance of VAMP721 over SYP121 for channel interaction in binary combination (Fig. 5C). However, when coexpressing KAT1 together with all three cognate SNAREs, we obtained K^+^ currents with *g*_max_ reduced by 23 ± 5% but with a small positive displacement of *V*_1/2_, compared with KAT1 alone (Fig. 5D; Supplemental Fig. S5). The results thus clearly showed a dominance of the Qa- and R-SNAREs in binary SNARE combinations on channel gating and their moderation of the current in tertiary combination. We return to these points later.

### K^+^ Channel Facilitates the SYP121 Conformational Changes for Oligomerization

**Figure 6.**
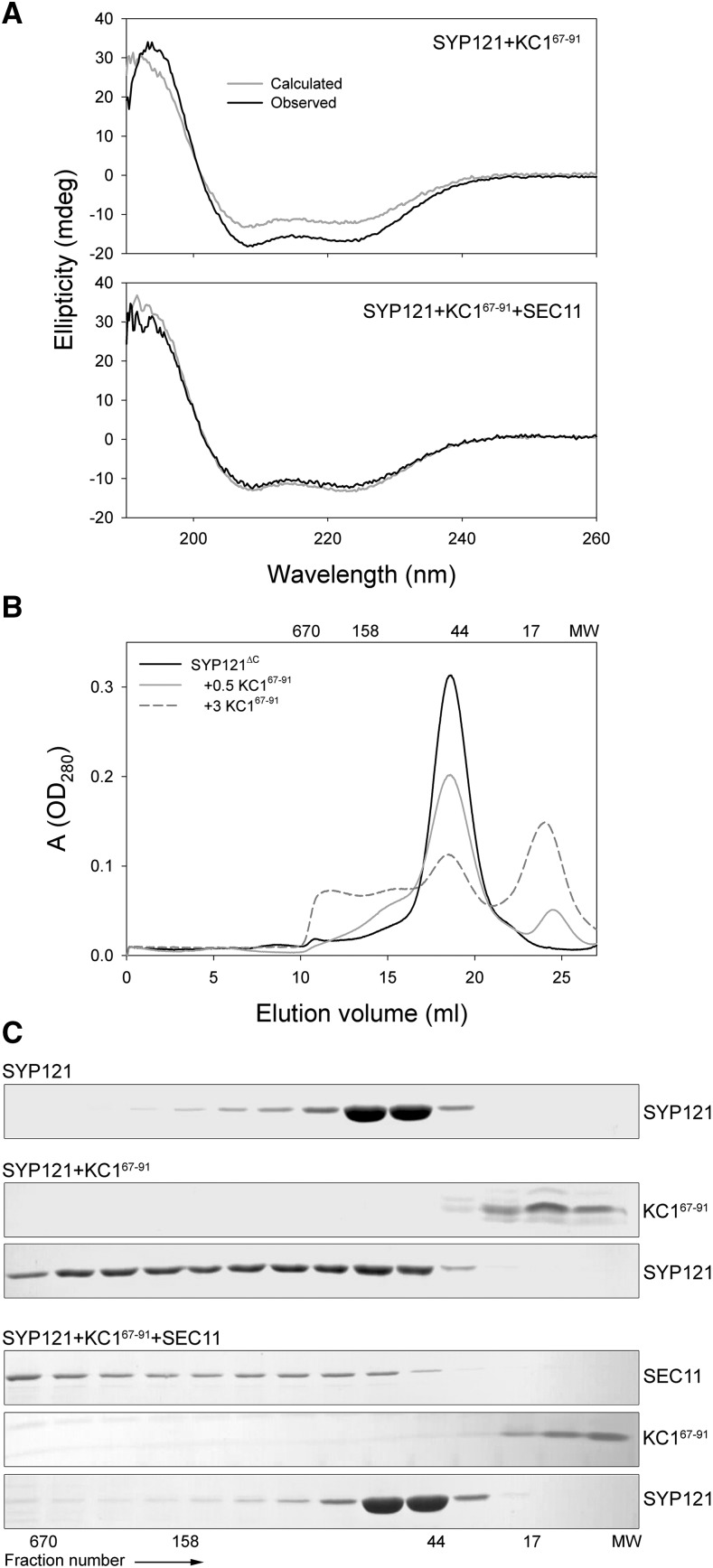
The K^+^ channel binding domain alters SYP121 structure to promote the open conformation. A, Far-UV CD spectra of KC1^67–91^ with SYP121^ΔC^-2PA mixed and incubated overnight in equimolar ratio shows roughly a 24% increase in spectral peak amplitudes between 200 and 230 nm above the arithmetical sum of individual spectra for the two proteins (top). This shift in peak amplitudes is suppressed on coincubation with SEC11 (bottom). Data are from one of three independent experiments, all of which yielded equivalent results with a mean of 28 ± 5% in peak amplitude above the spectral sum. Similar results were obtained with KAT1^1–63^ (see Supplemental Fig. S6). B, Size-exclusion chromatography of SYP121^ΔC^-2PA incubated overnight at 4°C alone and on coincubation with KC1^67–91^. In the closed conformation, SYP121^ΔC^-2PA eluted as a single peak corresponding to a molecular weight around 48 kD. When mixed with increasing amounts of the channel peptide, this peak was reduced in favor of a higher-molecular weight shoulder and peak spanning to near 650 kD, consistent with transition to the Qa-SNARE open conformation and formation of multimers. Molecular weights of standards are indicated above the columns. C, SDS-PAGE analysis of the gel filtration eluate collected in 0.75-mL fractions for fractions 11 to 24 collected after overnight incubations of SYP121^ΔC^-2PA alone, with KC1^67–91^, and with KC1^67–91^ together with SEC11. Note the shift of the SYP121^ΔC^-2PA to higher-molecular weight fractions in the presence of the channel peptide and its suppression when SEC11 is included in the incubation mix. Molecular weights of standards are indicated below the columns.

Previously we hypothesized that K^+^ channel binding with SYP121 facilitates transition of the Qa-SNARE to the open conformation for assembly with its cognate SNARE partners (Karnik et al., 2017). A central question behind this idea is whether channel binding alters SYP121 conformational structure. To address this question, we used circular dichroism (CD) to compare the secondary structural content of equal concentrations of SYP121^ΔC^ and KC1^67–91^ alone and in mixture. Equivalent experiments were carried out with the KAT1^1–63^ peptide. The far-UV CD spectrum typically shows maxima and minima between 200 and 230 nm that reflect this structural content. Thus, we anticipated that the spectrum of each mixture would be identical to the arithmetic sum of the spectra for channel peptide and SYP121^ΔC^ alone, provided that neither affected the structure of the other. In fact, the CD spectra of SYP121^ΔC^ with KAT1^1–63^ yielded a 15 ± 4% enhancement in the minima at 208 and 222 nm (Supplemental Fig. S6) and a 28 ± 5% enhancement with the KC1^67–91^ peptide (Fig. 6A), suggesting that interaction with the channel peptides was sufficient to alter the Qa-SNARE and channel peptide conformations. We also added KC1^67–91^ together with SEC11. In this case, we observed no substantive change in the CD spectrum beyond that calculated from the sum of the individual peptides (Fig. 6A), indicating that the SM protein suppressed the effects of the channel on SYP121.

Several mammalian and yeast Qa-SNAREs are known to form homo-oligomers in the open conformation when the N-terminal Habc α-helices release the so-called H3 domain, exposing it for SNARE binding (Fasshauer et al., 1998b; Lerman et al., 2000; Misura et al., 2001; Sieber et al., 2006, 2007). SYP121 is no exception to this mode of interaction (Kargul et al., 2001). As an independent test of the Qa-SNARE conformation, we therefore asked whether the channel peptide might promote SYP121^ΔC^ homo-oligomerization. We incubated SYP121^ΔC^ in solution alone and together with KC1^67–91^, thereafter carrying out size-exclusion chromatography to determine the resulting elution patterns. The results (Fig. 6, B and C) showed a single prominent peak when SYP121^ΔC^ passed through the column on its own. With increasing additions of the KC1^67–91^ peptide, a reduction in the size of this peak was evident with the appearance of shoulders and peaks at higher molecular weight retention volumes, the appearance of SYP121^ΔC^ in the early-eluting fractions (Fig. 6C) along with a peak near 13 kD that corresponded with the channel peptide (Fig. 6, B and C). The lack of obvious coelution of the channel peptide in this case is consistent with its lower component fraction in incubation, suggesting a catalytic role in promoting the Qa-SNARE open conformation and exclusion of the channel peptide from the SYP121^ΔC^ oligomers. This interpretation is also consistent with the effects of SEC11. When the SM protein was included, we observed a loss in Qa-SNARE oligomers but no change in the channel peptide distribution (Fig. 6C). Thus, these findings strongly suggest that the channel is able to catalyze conformational changes in SYP121^ΔC^ that expose the H3 domain, facilitating homo-oligomerization of the Qa-SNARE, but in the presence of SEC11, these conformational changes are suppressed (Fasshauer et al., 1998a).

### SEC11 Coordinates SNARE Binding Differentially with Channel Gating

SEC11 is an important regulator of SYP121-mediated vesicle traffic (Karnik et al., 2013b, 2015). Like other SM proteins, SEC11 binds with SYP121 in both the closed and open conformations via two different domains of the SM protein. Binding of a minor cleft of SEC11 with the N terminus of SYP121 overlaps with the K^+^ channel-binding motif F^9^xRF (Grefen et al., 2010), but SEC11 binding also occurs in association with a separate domain independent of channel binding with the Qa-SNARE and associated with the R^20^R^21^ motif of SYP121 (Zhang et al., 2019). SEC11 itself also binds the K^+^ channels, the binding motif overlapping with that for channel-SYP121 binding (Figs. 1 and 2). These are characteristics expected of a three-way binding exchange between SYP121, SEC11, and the K^+^ channels. Thus, we wanted to know whether the channel peptide and SEC11 compete for or facilitate SYP121 binding, and whether the SM protein might have complementary effects on binding between the other cognate SNARE partners.

**Figure 7.**
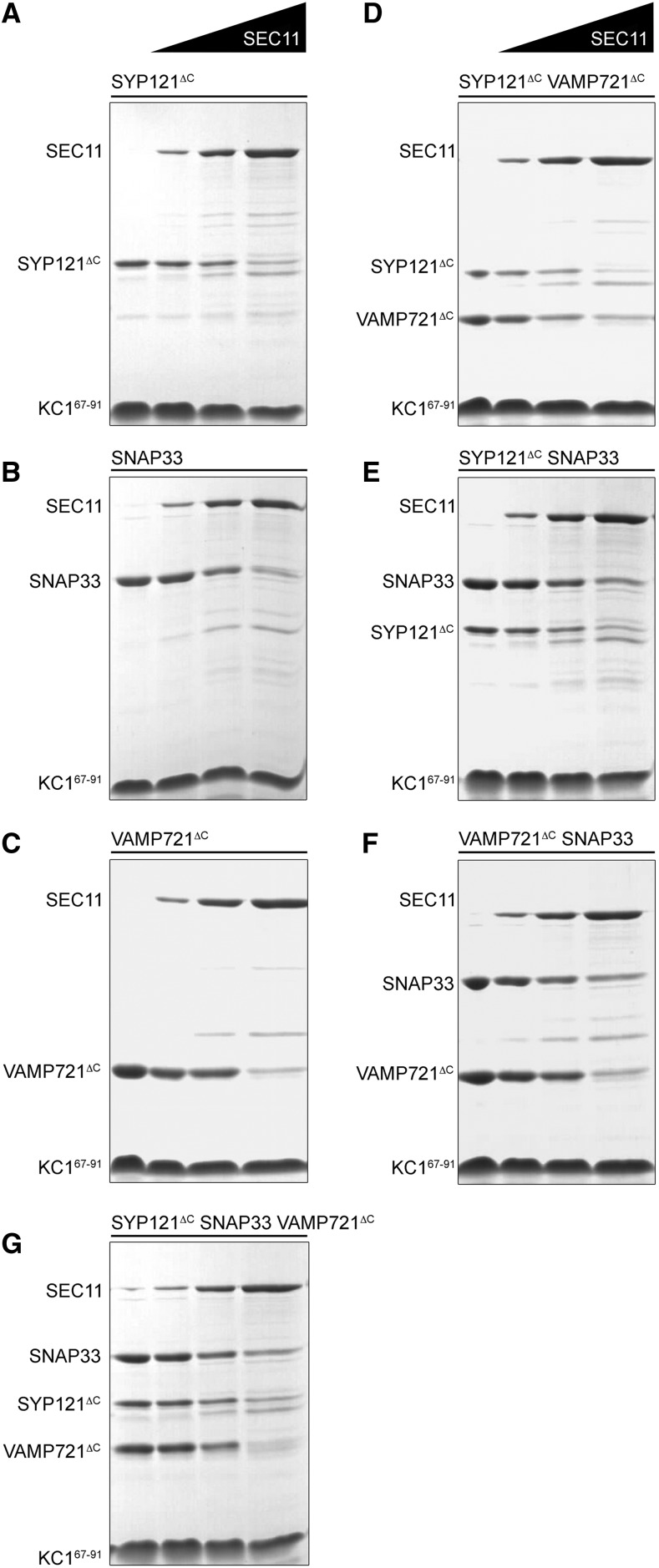
SEC11 competes differentially with KC1^67–91^ for cognate SNARE binding. Pulldown assays with KC1^67–91^ alone and with 0.3-, 1-, and 3-fold molar excess of SEC11 together with each of the cognate SNAREs, as indicated (top). Binding was tested with the SNAREs singly (A–C), in binary (d–F), and in tertiary (G) combinations, as indicated. Incubations were carried out overnight at 4°C before separation. Shown are Coomassie-stained SDS-PAGE gels of the bound proteins from one of three independent experiments in each case. Similar results were obtained with the KAT1^1–63^ peptide (Supplemental Fig. S7), and the results of all experiments are summarized in Figure 8.

**Figure 8.**
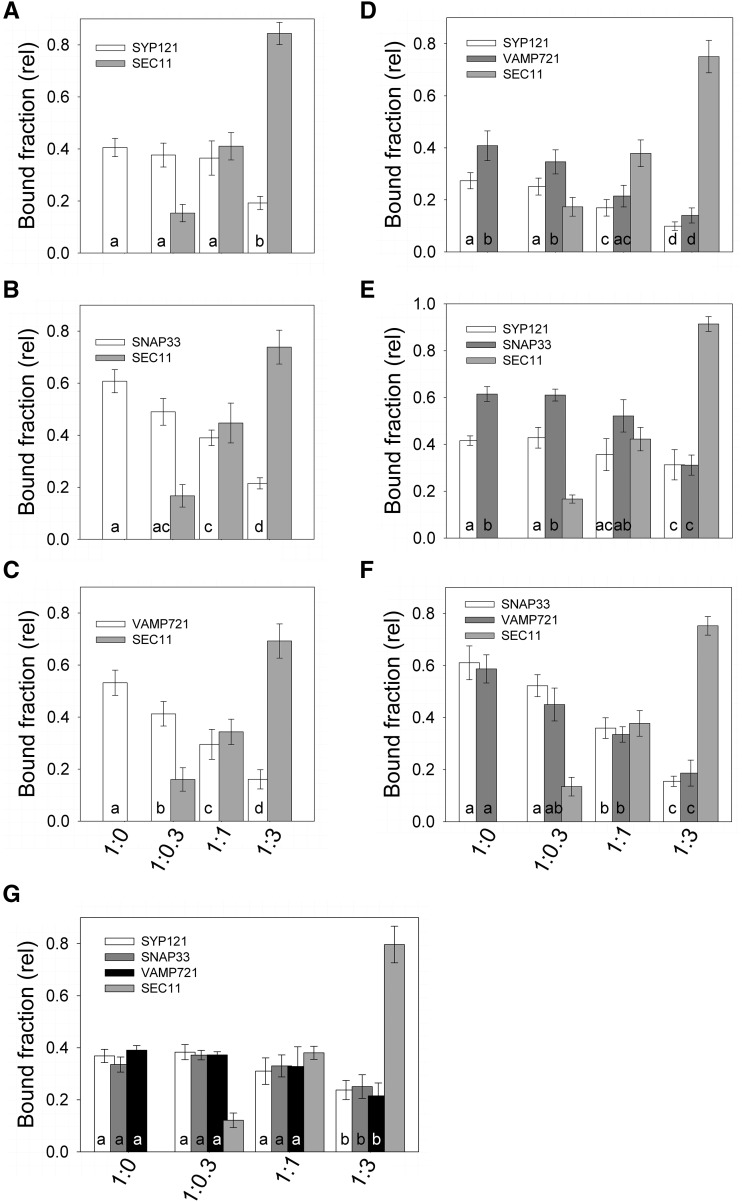
Bound fraction analysis of KC1^61-97^ and KAT1^1-63^ pulldowns from all six independent experiments alone and with SEC11 additions. Binding was with the SNAREs singly (A–C), in binary (D–F) and in tertiary (G) combinations as indicated and includes the results of experiments shown in Figure 7 and Supplemental Figure S7. Data are means ±SE of the ratios of SNAREs and SEC11 bound to K^+^ channel peptide. Letters indicate statistical differences at P<0.05.

To examine the role of SEC11 in the SNARE channel, we used pulldown experiments as before, employing the Strep-Tactin-immobilized channel peptides and cognate SNAREs without and with SEC11 in molar ratios of 0.3-, 1-, and 3-fold the concentration of SNAREs. Adding SEC11 to the cognate SNAREs individually (Fig. 7, A–C) resulted in a corresponding loss in recovery of the SNAREs, notably with VAMP721 and SNAP33. Adding SEC11 to binary SNARE combinations (Fig. 7, D–F) showed an enhanced recovery of SNAP33 with SYP121^ΔC^ compared to on its own. By contrast, in binary combination with VAMP721^ΔC^, increasing SEC11 showed a reduced recovery of both SNAREs. Finally, combining the cognate SNAREs (Fig. 7G) led to a recovery of all three SNAREs in constant ratio with increasing SEC11, although at high concentration of the SM protein the total recovery was reduced. Similar results were obtained in each of three independent experiments with KAT1^1–63^ (see also Supplemental Fig. S7), and the combined results are summarized in Figure 8. These findings suggest that SEC11 affects differentially the association of the channel with SYP121^ΔC^ and with VAMP721^ΔC^, it favors SNAP33 together with SYP121^ΔC^, and it stabilizes the SNARE complex when all three cognate partners are present.

**Figure 9.**
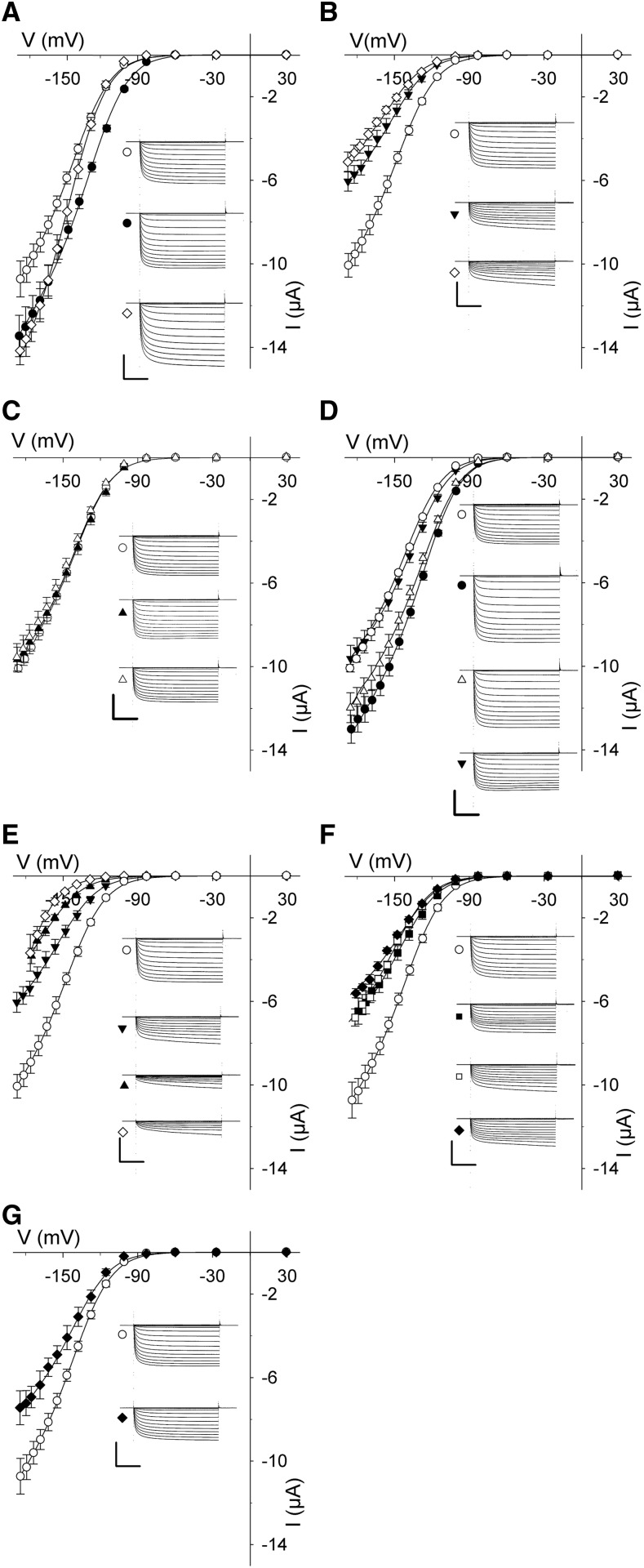
SEC11 promotes KAT1 activity with SYP121, but not with VAMP721 or SNAP33. Mean steady-state voltage curves recorded under voltage clamp from oocytes coexpressing the KAT1 channel alone and with combinations of the cognate SNAREs SYP121, VAMP721, and SNAP33 with SEC11. Data in every case are measurements from at least five independent experiments and are fitted jointly by nonlinear least-squares regression to the Boltzmann function of Equation 1. Each panel includes representative current traces cross-referenced by symbol. Representative immunoblot analyses and fitting results are included in Supplemental Figure S5. Data for KAT1 alone and with the individual SNAREs are reproduced in selected panels for reference. Water-injected controls yielded currents of <300 nA at all voltages and are omitted for clarity. Scales represent 1 s (horizontal) and 6 μA (vertical). A, KAT1 alone (○) and coexpressed with SYP121 (●) and SYP121 and SEC11 (♢). B, KAT1 alone (○) and coexpressed with VAMP721 (▼) and VAMP721 and SEC11 (♢). C, KAT1 alone (○) and coexpressed with SNAP33 (∆) and SNAP33 and SEC11 (▲). D, KAT1 alone (○) and coexpressed with SYP121 (●), SYP121 and SNAP33 (∆), and SYP121, SNAP33, and SEC11 (▼). E, KAT1 alone (○) and coexpressed with VAMP721 (▼), VAMP721 and SNAP33 (♢), and VAMP721, SNAP33, and SEC11 (▲). (F) KAT1 alone (○) and coexpressed with VAMP721 (▪), SYP121 and VAMP721 (□), and SYP121, VAMP721, and SEC11 (♦). G, KAT1 alone (○), and coexpressed with SYP121, VAMP721, SNAP33, and SEC11 (♦).

To assess the consequences of SEC11 in combination with the cognate SNARE partners in vivo, again we recorded KAT1 current under voltage clamp on coexpression with SEC11 and the several SNARE combinations in *Xenopus* oocytes. Figure 9 summarizes the mean steady-state current-voltage curves recorded from at least five independent experiments, in each case with representative current traces for each combination of SNARE components as well as the fitted parameters for gating (Supplemental Fig. S5). Immunoblots confirming protein coexpression are included in Supplemental Fig. S5. Expressing KAT1 with SEC11 alone had no significant effect on the K^+^ current or its gating characteristics, even though the SM and channel proteins bound in vitro (Fig. 3; Supplemental Fig. S3). Similarly, expressed in combination with VAMP721 or with SNAP33, we found that SEC11 protein had little or no effect on channel current and gating (Fig. 9, B and C); in each case, the current characteristics and fitted values for *V*_1/2_ (Supplemental Fig. S5) were indistinguishable from those with the SNAREs alone. Similarly, coexpression of SEC11 with the SNARE combinations with SNAP33 and SYP121 with VAMP721 had no appreciable effect additional to that of the SNAREs alone (Fig. 9, D and F; Supplemental Fig. S5). Expressing SEC11 with SYP121 and SNAP33 recovered a current similar to that of KAT1 alone, albeit with a small but significant positive shift in *V*_1/2_ (Fig. 9D). However, expressed with VAMP721 and SNAP33, SEC11 strongly suppressed the channel current (Fig. 9E), shifting *V*_1/2_ to extreme negative voltages (Supplemental Fig. S5).

Of particular interest, we also found that expressing SEC11 with SYP121 alone increased g_max_ and yielded a modest negative-going shift in *V*_1/2_ when compared with the effect of coexpressing SYP121 alone (Fig. 9A), an effect that was essentially complete at a SEC11:SYP121 molar ratio of unity (Supplemental Fig. S8). In this case, the result was a steady-state current that followed the KAT1 current at more positive voltages and the KAT1+SYP121 current at more negative voltages, indicating a pronounced voltage dependence to the action on gating of SEC11 with the Qa-SNARE, as if the efficacy of the SM protein is suppressed with negative-going voltage. Finally, when coexpressing all three SNAREs with SEC11, we found that the KAT1 current was moderated (Fig. 9G), much as in the absence of SEC11 (Fig. 5D) and with no significant change in *V*_1/2_ (Supplemental Fig. S5). These data, together with the biochemical analyses above, indicate a critical role for SYP121 interactions with the K^+^ channels and their coordination with SEC11 in triggering SNARE complex assembly. We return to this point below.

## DISCUSSION

SNARE assembly, including that of SYP121, VAMP721, SNAP33, and the SM protein SEC11 (Lipka et al., 2007; Bassham and Blatt, 2008; Karnik et al., 2013b, 2015, 2017), engages a highly concerted sequence of conformational changes and binding and debinding events that culminate in vesicle intercalation with the plasma membrane (Jahn and Scheller, 2006; Archbold et al., 2014; Baker and Hughson, 2016). Uniquely in plants, substantial evidence indicates that these events are tightly coordinated with noncanonical interactions between subsets of plasma membrane SNAREs and voltage-gated K^+^ channels, the interactions promoting secretory traffic together with alterations in channel gating that facilitate K^+^ uptake (Honsbein et al., 2009; Grefen et al., 2015; Lefoulon et al., 2018; Zhang et al., 2019). How the channel interactions are integrated within the process of SNARE assembly has remained an open question. All the more challenging are observations that Qa- and R-SNARE binding have opposing effects on K^+^ channel gating, K^+^ uptake, and growth (Grefen et al., 2010; Zhang et al., 2015), thus posing questions about which binding mode dominates within the temporal cycle of SNARE complex assembly.

We have explored these questions by isolating the SNARE and channel components to study their interactions in vitro and the consequences for channel gating in vivo in the defined heterologous system of the *Xenopus* oocyte. Remarkably, we find that all three cognate SNAREs, SYP121, VAMP721, and SNAP33, bind with the N-terminal cytosolic domain of the K^+^ channels KAT1 and KC1 through the conserved linear motif RYxxWE situated at the cytosolic base of the first transmembrane α-helix of the channel VSD. This motif overlaps also in binding of the SM protein SEC11, implying a highly concerted sequence of binding exchanges with the K^+^ channels. Three lines of evidence suggest that channel binding is dominated by the Qa-SNARE SYP121 and is coordinated through a binding exchange with SEC11 and stabilized by SNAP33. (1) Only SYP121 and VAMP721 exhibit a strong competition for channel binding, and only in binary combination without SNAP33; with SNAP33 present, all three cognate SNAREs were recovered in pulldown with the channel peptide. (2) Titrated against SEC11, channel peptide binding with SNAP33 and VAMP721 was suppressed, but binding with SYP121 was not, either in binary combination with SNAP33 or in tertiary combination with all three cognate SNAREs. (3) Finally, SYP121 association with the channels, alone and with SEC11, showed alterations in Qa-SNARE conformation and channel gating that are consistent with a three-way exchange between the partners that was affected by SNAP33. These findings are most easily understood in the context of channel binding that coordinates initially with SEC11 to drive SYP121 from its closed to open conformation, enhancing channel activity for K^+^ flux and thereafter tempering K^+^ uptake with tertiary SNARE assembly. They indicate, too, that SNAP33 is important in moderating channel binding with SYP121 and VAMP721. Thus, we suggest that the primary action of the K^+^ channels is in a handover between SEC11 and SYP121 that allows the conformational transition of the Qa-SNARE for binding with its cognate SNARE partners. We also suggest that channel association with the R-SNARE alone and in combination with SNAP33 may serve as a fail-safe, precluding interactions that might otherwise disengage ion transport and secretory traffic.

### SNAP33 Facilitates K^+^ Channel Activity with SNARE Complex Assembly

Most surprising was the finding that K^+^ channel binding for all three cognate SNAREs was isolated to a short region of the channel N terminus and incorporated a common motif (Figs. 1 and 2; Supplemental Figs. S1 and S2). This motif is located at the cytosolic face of the plasma membrane immediately preceding the first transmembrane α-helix of the channel VSD. The VSD structure itself is highly conserved among voltage-gated (Kv) K^+^ channels across kingdoms and it confers a voltage dependence to channel opening, including that of KAT1 (Lefoulon et al., 2014), by coupling movement of the VSD within the membrane to the channel pore (Palovcak et al., 2014). Significantly, the RYxxWE is largely conserved among a subset of Kv channels of land plants, is critical for binding SYP121 that confers a voltage dependence on secretory traffic (Grefen et al., 2015), and modulates the gating of channels assembled of the KAT1 and KC1 subunits (Honsbein et al., 2009; Grefen et al., 2010; Lefoulon et al., 2018).

That this motif is common also to binding with VAMP721 and SNAP33 raises obvious questions of competition for channel binding and connections to the voltage sensitivity of channel gating. Indeed, pulldown analysis with the K^+^ channel N termini as bait showed that SYP121 and VAMP721 compete for binding (Fig. 4; Supplemental Fig. S4). Furthermore, functional analysis using the KAT1 channel current as a proxy for SNARE interaction showed opposing effects on gating, with a dominance of VAMP721 over SYP121 (Fig. 5; Supplemental Fig. S5). The latter findings extend those of previous electrophysiological studies implicating the apposition of the Qa- and R-SNAREs on the K^+^ channels (Honsbein et al., 2009; Grefen et al., 2015; Zhang et al., 2015). No such action was evident with SNAP33 alone, however, either in pulldown or electrophysiological experiments. From these data, we cannot rule out binary binding populations of the channel bait with SNAP33 and its cognate SNAREs or their possible displacements of SNAP33 from action on the KAT1 channel in vivo. However, such explanations are difficult to reconcile with the observations that the channel N termini recovered all three cognate SNAREs in roughly a 1:1:1 ratio when added together (Fig. 4; Supplemental Fig. S4). Furthermore, SNAP33 in binary combination with VAMP721 enhanced the effect of the R-SNARE in suppressing the KAT1 current, while its inclusion together in tripartite combination abolished the apparent competition of SYP121 and VAMP721 for KAT1 gating and tempered channel activity in vivo (Figs. 5 and 9). These findings argue instead that the channels preferentially coordinate with all three SNAREs as they assemble in complex, and they suggest that SNAP33 acts to moderate the functional impact of the Qa- and R-SNAREs, favoring a relaxation of K^+^ flux through the channels on final SNARE complex assembly.

It is worth noting that the actions of SNAP33, like those of SYP121 in vivo (Honsbein et al., 2009; Lefoulon et al., 2018), differ from those of its animal counterpart, SNAP25. Binding and functional studies have suggested that in mammalian cells, SNAP25 may suppress K^+^ and Ca^2+^ channel gating either alone or together with its cognate Qa-SNARE (MacDonald et al., 2002; Leung et al., 2007; Weiss and Zamponi, 2012). These effects are modest, and there remains some controversy as to whether the actions of SNAP25 are associated with its cleavage products (He et al., 2008). Furthermore, assembly of SNAP25 and its cognate SNAREs Syntaxin 1A and VAMP2 abolishes K^+^ channel binding and eliminates SNARE actions on channel activity (Tsuk et al., 2008), raising a basic question about its functional significance in the temporal coordination of membrane excitability.

### SEC11 Complements K^+^ Channel Binding for SYP121 Conformational Transitions

A further dimension to SNARE-channel coordination is added with our discovery that SEC11 binds the K^+^ channels. Like other SM proteins (Südhof and Rothman, 2009; Archbold et al., 2014), SEC11 binding with the SYP121 N terminus is thought to serve in part as an anchor point for the SM protein during Qa-SNARE transit from the closed to the open state (Karnik et al., 2013b, 2015). SEC11 binding with SYP121 is subject to the highly conserved Phe-9 that situates within the F^9^xRF motif for the K^+^ channel (Grefen et al., 2010). SEC11 also binds the Qa-SNARE N terminus via a second site centered on a unique R^20^R^21^ motif independent of channel binding, an observation that has led to the suggestion of a binding exchange and displacement of the SM by the channel proteins (Zhang et al., 2019). This concept of an exchange cascade now gains further support. We observed that SEC11 interacts with the channels through a motif overlapping with the RYxxWE SNARE-binding motif (Fig. 2; Supplemental Fig. S2) and, furthermore, that it is able to compete for channel binding with each of the cognate SNAREs individually but with reduced efficacy with the binary combination of SYP121 and SNAP33 (Figs. 4, 7, and 8; Supplemental Figs. S4, S7, and S8). Furthermore, SEC11 interaction with KAT1 showed a pronounced voltage dependence when SYP121 was present (Supplemental Fig. S8). We also found that the channel N-termini facilitated SYP121 homo-oligomerization, a common feature of open Qa-SNAREs that is enhanced in the soluble domain and is suppressed by SM protein binding (Dawidowski and Cafiso, 2013), for SYP121 by SEC11 (Fig. 6; Supplemental Fig. S6). These findings indicate that the channels compete with SEC11 to promote transit to the open Qa-SNARE conformation, which, when the Qbc-SNARE SNAP33 is present, may stabilize SYP121 binding with the K^+^ channels.

Features of the K^+^ currents offer additional insight into possible mechanisms of a binding exchange with the SNAREs. Unlike the pulldown experiments, channel currents recorded under voltage clamp speak to the interactions of the full-length channel protein in the membrane and the impact of membrane voltage. These data (Figs. 5 and 9; Supplemental Fig. S8) show that SEC11 had little impact on KAT1 channel gating with VAMP721 alone. However, when coexpressed with SYP121, SEC11 promoted a KAT1 current that approximated the characteristics of KAT1 alone near and positive of −120 mV and was augmented like that of KAT1 with SYP121 alone at more negative voltages. In other words, SEC11 associated functionally with the KAT1-SYP121 combination such that the functional impact of its binding was suppressed at more negative voltages. At present, it is not possible to resolve the voltage dependence of any of these complex interactions directly in vitro. Nonetheless, the current characteristics suggest that membrane hyperpolarization favors K^+^ channel association with SYP121 binding, overcoming SEC11 action, and that the voltage dependence introduced by SEC11 is moderated when SNAP33 is also present. These findings are entirely consistent with the enhancement of K^+^ uptake and voltage dependence conferred on traffic by the channel VSD (Honsbein et al., 2009; Grefen et al., 2015).

How might these characteristics of SNARE and SM binding and channel gating be understood in the context of SNARE assembly? Secretory traffic mediated by SYP121 shows a substantial dependence on membrane voltage coordinated through the channel VSD with K^+^ flux enhancement (Honsbein et al., 2009; Grefen et al., 2015) and is uniquely subject to SEC11 (Karnik et al., 2013b, 2015). We note, too, that SEC11 binding altered channel voltage sensitivities in association with SYP121, but not in association with VAMP721 alone (Figs. 5 and 9). Thus, it follows that K^+^ channel binding with the R-SNARE “locks out” further transition of the complex to suppress secretory traffic and K^+^ flux. This interpretation accords with previous findings that the *vamp721* mutant showed a modest enhancement of growth and that growth was suppressed on overexpressing VAMP721 (Zhang et al., 2015, 2017). We speculate that binary interactions centered around the R-SNARE reflect a fail-safe mechanism that prevents departure from the temporal sequence of binding transitions that lead to vesicle fusion.

**Figure 10.**
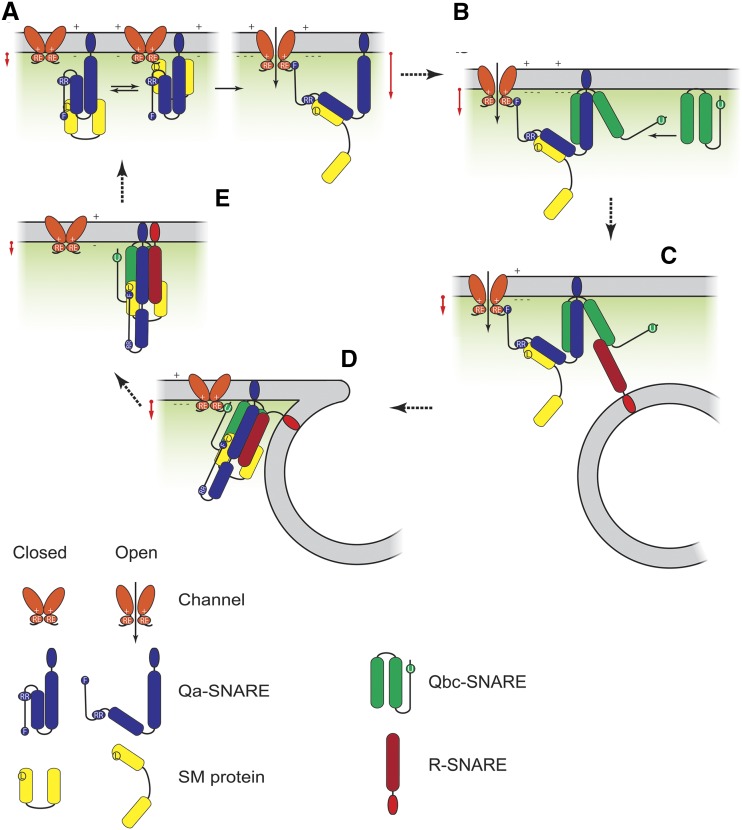
K^+^ channels KAT1 and KC1 facilitate a binding exchange with SEC11 to promote SNARE assembly for vesicle fusion. The SM protein SEC11 (A) holds SYP121 in the closed conformation through its major cleft and also binds via L^128^ (circled L) with the Qa-SNARE N-terminal motif centered on residue F^9^ (circled F; Karnik et al., 2013b, 2015). SYP121 harbors a second SEC11-binding site centered on its R^20^R^21^ motif (circled RR) that does not affect channel binding (Zhang et al., 2019). SEC11 interacts with the K^+^ channels through the RYxxWE motif (circled RE) and, with membrane hyperpolarization (+, −), undergoes a three-way binding exchange between SEC11 and SYP121 that culminates with binding through the two motifs, the channel RYxxWE motif (RE) and the Qa-SNARE FxRF motif (F), thereby conferring an apparent voltage dependence on the channel-SM protein interaction with SYP121 (Supplemental Fig. S8) and enhancing channel gating and activity (Honsbein et al., 2009; Grefen et al., 2010, 2015). The Qbc-SNARE SNAP33 (B) stabilizes the complex of SYP121, SEC11, and the K^+^ channel to moderate the open channel, Qa-SNARE, and SM protein conformations (Fig. 9). Recruiting the R-SNARE VAMP721 (C) facilitates final assembly of the SNARE core complex and transfer of channel binding (D) to SNAP33 (unknown site [circled U]) while relaxing channel gating and conductance (Fig. 9). Finally, disengaging channel binding with the cis SNARE complex (E) is followed by SNARE complex disassembly. Red arrows by each step in the cycle indicate the nominal channel activity for K^+^ uptake and its anticipated enhancement with the open conformation of SYP121 prior to assembly with VAMP721. Note that binding motifs on SEC11 and SNAP33 for the K^+^ channels are yet to be determined. The binding motif on SEC11 associated with the SYP121 R^20^R^21^ motif is also unknown. The time-averaged K^+^ flux needed to support cell expansion, for example during stomatal opening, is equivalent to a current of 1 to 3 μA cm^−2^ on a cell surface basis and is readily accommodated by KAT1 at voltages near −100 to −120 mV in vivo (Jezek and Blatt, 2017).

By the same token, we note that SYP121, like other Qa-SNAREs (Südhof and Rothman, 2009; Rizo and Südhof, 2012; Archbold et al., 2014), is thought to be held in closed conformation by SEC11, with the SNARE-binding (H3) domain occluded. Thus, debinding of the SEC11 major cleft and opening of the Qa-SNARE is an early step driving SNARE assembly and vesicle fusion (Karnik et al., 2013b, 2015). Here, channel binding with SYP121 is important for the transition of SYP121 to the open form, likely displacing SEC11 binding with the Qa-SNARE through a three-way binding exchange that promotes the K^+^ current and culminates with SYP121 in the open conformation, bound with the channel, SNAP33, and SEC11, the latter via its minor cleft (Karnik et al., 2013b, 2015). Our results are most easily explained in the context of such a model (Fig. 10). The data are consistent with a stabilization of this state through its association with SNAP33, thereby priming the Qa- and Qbc-SNAREs for final assembly with VAMP721. The model is also consistent with an enhanced rate of K^+^ uptake that is temporally coordinated with vesicle fusion. Indeed, even a 2- to 3-fold increase in K^+^ flux, as implicated for the current enhancement with SYP121 (Figs. 5 and 9), is likely to be sufficient to support K^+^ with cell expansion if it is maintained for 20% of the cycle period, as illustrated in Figure 10. In effect, our interpretation implies a role for the K^+^ channels analogous to that of MUNC13 in orchestrating the binding release of the SM protein Munc18 from Syntaxin 1A for secretion in mammalian neurons (Ma et al., 2013; Wang et al., 2019). It accords, too, with previous studies demonstrating that the K^+^ channels accelerated secretory traffic dependent on binding with the channel VSD (Grefen et al., 2015), and with the finding that, once bound, the Qa-SNARE interaction with the channel is stable relative to the conformational lifetimes of the channel (Lefoulon et al., 2018).

In conclusion, we propose that binding exchange between SEC11 and the K^+^ channels with SYP121 represents the critical starting point that regulates the final steps to vesicle fusion while promoting K^+^ uptake. Such a mechanism ensures the timely engagement of the Qa-SNARE with membrane voltage as a common measure of the transport activities driving solute flux for cell expansion. We suggest, too, that K^+^ channel association with the cognate SNARE SNAP33 is important to stabilize the nascent Q-SNARE complex at the plasma membrane in preparation for final assembly with the R-SNARE and vesicle fusion. Thus, SEC11 and K^+^ channel binding unite to define a unique, temporal sequence that juxtaposes ion transport with enhanced secretory vesicle traffic.

## MATERIALS AND METHODS

### Molecular Biology

*Escherichia coli* strains of BL21(DE3) were used as a host for protein expression (Invitrogen) and constructs were cloned into pETDuet (Carpp et al., 2006) and pRSFDuet (Addgene) expression vectors. We used a classical cloning strategy to generate expression cassettes combining the multiple flag tags (Flag_3_ and Flag_6_) of the vectors with selections of 6His, 2Protein A (2PA), or Strep-II affinity tags on both upstream and downstream positions. Constructs tagged with 2PA were those described previously (Karnik et al., 2013b). The cassettes were integrated into the backbone of pETDuet and pRSFDuet using *Nco*I and *Not*I restriction sites. The resultant vectors were verified by sequencing and named pETDuet-Flag and pRSFDuet-Flag. Open reading frames for SYP121^ΔC^, VAMP721^ΔC^, SNAP33, SEC11, KC1^67–91^, and KAT1^1–63^ were cloned into pETDuet, pETDuet-Flag, and pRSFDuet-Flag by classical cloning with the primers and restriction sites listed in Supplemental Table S1.

For split-ubiquitin assays and expression in *Xenopus laevis* oocytes, entry vector constructs of SYP121, SYP121^ΔC^, VAMP721, VAMP721^ΔC^, SNAP33, and SEC11, as well as KC1 and KAT1, their truncations, and single-site mutations were generated in pDONR207 as described previously (Grefen et al., 2010, 2015; Zhang et al., 2015, 2018, 2019; Lefoulon et al., 2018) and used in Gateway reactions with LR Clonase II (Life Technologies) to generate the corresponding destination constructs. Single-site mutations were generated by site-directed mutagenesis (Karnik et al., 2013a; Grefen et al., 2015; Zhang et al., 2017). Primers for point mutations were designed by SDM-Assist software (Karnik et al., 2013a) to include unique “silent” restriction sites along with the desired mutation for later identification by restriction endonuclease digestion. Gateway entry clones and destination clones were amplified using Top10 cells (Life Technologies) with 20 mg/L Gentamicin or Kanamycin for entry clones and 100 mg/L Spectinomycin for destination clones. All mutations were sequenced and validated.

For electrophysiological experiments, gateway entry vectors carrying the coding sequences for KAT1, SYP121, VAMP721, SNAP33, and SEC11 transferred in pGT‐Dest‐myc for KAT1, pGT-Dest-HA for VAMP721, and pGT-Dest for SYP121, SNAP33, and SEC11 by LR reaction as described previously (Lefoulon et al., 2014, 2018; Grefen et al., 2015; Karnik et al., 2015).

### mbSUS Assays

mbSUS assays were performed as described before (Karnik et al., 2013b; Grefen et al., 2015; Horaruang and Zhang, 2017). Bait constructs were transformed into yeast strain THY.AP4 and prey constructs were transformed into THY.AP5. Ten to 15 yeast colonies were selected and inoculated into selective media (complete supplement mixture [CSM]_-LM_ for THY.AP4 and CSM_-MTU_ for THY.AP5) for overnight growth at 180 rpm and 28°C. Liquid cultures were harvested and resuspended in yeast peptone dextrose (YPD) medium. Yeast mating was performed in sterile PCR tubes by mixing equal aliquots of yeast containing bait and prey constructs. Aliquots of 5 µL were dropped on YPD plates and incubated at 28°C overnight. Colonies were transferred from YPD onto CSM_-LMTU_ plates and incubated at 28°C for 2 to 3 d. Diploid colonies were selected and inoculated in liquid CSM_-LMTU_ media and grown at 180 rpm 28°C overnight before harvesting by centrifugation and resuspension in sterile water. Serial dilutions at OD_600_ 1.0 and 0.1 in water were dropped (5 µL per spot) onto CSM_-AHLMTU_ plates with added Met. Plates were incubated at 28°C and images were taken after 3 d. Yeast was also dropped on CSM_-LMTU_ control plates to confirm mating efficiency and cell density, and growth was imaged after 24 h at 28°C. To verify expression, yeast was harvested and extracted for protein immunoblot analysis using commercial hemagglutinin (HA) antibody for prey and commercial VP16 antibody (Abcam) for the bait.

### Recombinant Protein Expression and Purification

pETDuet-Flag and pRSFDuet-Flag plasmids containing each construct were chemically transformed into *E. coli* BL21(DE3) cells. After incubating overnight at 37°C on lysogeny broth (LB) 1% (w/v) agar plates, single colonies were picked and inoculated into 5 mL LB medium with 100 μg/mL Ampicillin and incubated at 37°C in a shaker at 180 rpm overnight. The 5 mL culture was transferred into 500 mL Terrific Broth (Thermo Fisher Scientific) medium supplemented with 50 μg/mL Ampicillin and incubated at 37°C and 180 rpm to an OD_600_ of 1.2. Thereafter, the culture was transferred to 18°C and induced with 1 mm isopropylthio-β-galactoside overnight before harvesting by centrifugation at 4,000*g* for 10 min. Pellets were suspended in 20 mL lysis buffer (50 mm Tris-HCl, 150 mm NaCl, 5% [v/v] glycerol, and 0.1% [v/v] Triton X-100, pH adjusted to 8.0) complemented with protease inhibitor (cOmplete, Mini, EDTA-free Protease Inhibitor Cocktail, Sigma-Aldrich). Cells were lysed by sonication for 5 min on ice. Lysates were centrifuged at 15,000*g* and 4°C for 30 min, and supernatants were stored at 4°C for further purification. Aliquots of the induced culture, supernatant, and pellet fractions were taken and mixed with an equal volume of 2× SDS sample buffer for SDS-PAGE analysis.

For purification of His-tagged proteins, the soluble fraction of cell lysate was loaded onto Ni-NTA resin (ThermoFisher) and washed with 15 column volumes of wash buffer (50 mm Tris-HCl, 150 mm NaCl, 21 mm imidazole, 5% [v/v] glycerol, and 0.1% [v/v] Triton X-100, pH 8.0) followed by two column volumes of elution buffer (50 mm Tris, 1 m NaCl, and 300 mm imidazole, pH 8.0). For purification of proteins with StrepII tags, the soluble fraction of cell lysate was loaded onto Strep-TactinXT Superflow (IBA-Life Sciences) affinity resin and washed with 15 column volumes of wash buffer (50 mm Tris-HCl, 150 mm NaCl, 5% [v/v] glycerol, and 0.1% [v/v] Triton X-100, pH 8.0) followed by two column volumes of elution buffer (50 mm Tris, 150 mm NaCl, and 2.5 mm desthiobiotin, pH 8.0). For purification of SYP121 with the Protein-A tag, the soluble fraction of cell lysate was loaded onto IgG-sepharose resin (GE Healthcare) and washed with 15 column volumes of wash buffer (50 mm Tris-HCl, 150 mm NaCl, 5% [v/v] glycerol, and 0.1% [v/v] Triton X-100, pH 8.0) followed by two column volumes of elution buffer (0.1 mm ammonium acetate, pH 3.4). In each case, the eluted samples were buffer-exchanged with 50 mm Tris, 5% [v/v] glycerol, and 150 mm NaCl, pH 8.0 using a HiPrep 26/10 desalting column (GE Healthcare) on FPLC (AKTA prime, GE Healthcare). Subsequently, the concentrations of purified proteins were determined by UV *A*_280_. Aliquots of all samples were mixed with an equal volume of 2× SDS sample buffer, heated at 90°C for 5 min, centrifuged at 14,000*g* for 5 min prior to SDS-PAGE, and visualized by staining with Coomassie brilliant blue.

### Pulldown and Gel Filtration Analysis

Pulldown experiments were performed using KC1^67–91^-3flag-StrepII and KAT1^1–63^-6flag-StrepII as baits immobilized on Strep-Tactin affinity resin. All reactions were carried out by adding prey proteins in the designated molar ratios and incubating the mixtures overnight at 4°C with gentle rotation. Pulldowns were analyzed after washing with the same buffer by eluting bait and bound proteins from the resin for SDS-PAGE and Coomassie staining.

Gel filtration chromatography was carried out using a Superdex 200 10/300 column (Generon) coupled to an AKTA purifier (GE Healthcare). Samples were loaded to the column equilibrated with 50 mm Tris-HCl and 150 mm NaCl, pH 8.0 buffer. The elution was performed at a flow rate of 0.5 mL/min and monitored at 220 nm, and fractions were collected for analysis by SDS-PAGE. Molecular weights were quantified in parallel chromatographic separations of the established marker proteins bovine thyroglobulin (670 kD), gammaglobulin (158 kD), chicken ovalbumin (44 kD), horse myoglobin (17 kD), and vitamin B12 (1.4 kD). Band intensities on SDS-PAGE were quantified using ImageJ v.1.52 (https://imagej.nih.gov/ij/) and standardized against known molar quantities of the same proteins. Bound fractions were calculated from these values relative to the eluted quantities of the relevant bait proteins.

### CD Spectroscopy

CD was determined using a Jasco J-810 spectropolarimeter (Jasco) at 20°C. Far-UV CD data were acquired in a quartz cuvette with a 0.02 cm pathlength using a scan rate of 50 nm/min and response time of 0.5 s. Spectra were collected over 185 to 260 nm with a 1-nm bandwidth after correction with blank spectra of the buffer. A final protein concentration of 0.3 mg/mL in 50 mm Tris, 150 mm NaCl, and 20 mm ammonium acetate, pH 8.0, was used in every case. Spectral data were analyzed using Jasco Spectra Analysis software.

### Electrophysiology and Immunochemistry

The complementary RNA of each construct was transcribed after template linearization using T7 mMessage mMachine (Ambion). Complementary RNA was confirmed by gel electrophoresis and injected into stage VI *Xenopus* oocytes, as before (Lefoulon et al., 2014, 2018; Grefen et al., 2015) in molar ratios as indicated. Electrical analysis was carried out over a period of 3 to 5 d postinjection. Whole-cell currents were recorded under voltage clamp using an Axoclamp 2B amplifier (Axon Instruments) and data were acquired after filtering with a 1 kHz (*f*_c_) 8-pole Bessel filter. Measurements were carried out with oocytes under continuous perfusion with osmotically balanced solutions containing 30 mm KCl, 70 mm NaCl, 1 mm CaCl_2_, 1.5 mm MgCl_2_, and 10 mm HEPES-NaOH, pH 7.4. Recordings were analyzed postacquisition using Henry IV software (Y-Science), as described previously (Honsbein et al., 2009; Grefen et al., 2010, 2015; Lefoulon et al., 2014, 2018).

Oocytes were collected individually after recording for immunoblot analysis. Immunoblots were performed as described before (Lefoulon et al., 2014, 2018; Grefen et al., 2015). Oocytes were homogenized in denaturing buffer (150 mm NaCl, 0.25% [w/v] SDS, 1% [w/v] NP-40, 1 mm EDTA, 1 mm NaF, 1.125 mm PMFS, and 50 mm Tris-HCl, pH7.4) at 10 µL/oocyte, and centrifuged at 425g for 5 min. One volume of loading buffer (4 M urea, 10% [w/v] SDS, 40 mm EDTA, 0.2% [v/v] Triton X-100, 0.1% [w/v] bromophenol blue, 20% [v/v] glycerol, 200 mm dithiothreitol, and 100 mm Tris-HCl, pH 6.8) was added to the supernatant, and samples were incubated for 30 min at 37°C before transfer to nitrocellulose and protein detection using the western Blot ECL Advance kit (GE Healthcare) with rabbit α‐myc (dilution 1:5000, Abcam) for KAT1, α‐HA (dilution of 1:4,000, Roche) for VAMP721, and with α‐SYP121 antibodies (dilution of 1:4,000), α‐SNAP33 (dilution of 1:2,000), and α‐sec11 (dilution of 1:2,000) accordingly (Assaad et al., 2001; Tyrrell et al., 2007; Honsbein et al., 2009; Karnik et al., 2013b; Lefoulon et al., 2018) after binding with secondary horseradish peroxidase‐coupled goat, anti‐rabbit antibodies (Abcam).

### Chemicals and Other Materials

The DNA polymerase KOD-plus-Neo was purchased from Toyobo Life Science (Osaka, Japan). Primer synthesis, DNA synthesis, and DNA sequencing were performed by IDT DNA, Genscript, and SourceBioscience, respectively. SDS-PAGE gels NuPAGE and prestained *M*_r_ marker were from Invitrogen. All of the chemicals and reagents were purchased from Sigma-Aldrich unless otherwise indicated. The plasmid mini prep kit was purchased from Qiagen.

### Statistical Analysis

Statistical analysis of experiments is reported as means ± se as appropriate with significance determined by Student’s *t* test or ANOVA in SigmaPlot v.11.2 (Systat Software). Joint nonlinear, least-squares fittings were carried out using the Marquardt-Levenberg algorithm of SigmaPlot v.11 (SPSS).

### Accession Numbers

Sequence data from this article can be found in the Arabidopsis Genome Initiative or GenBank/EMBL data libraries under the following accession numbers: At5g46240 (KAT1), At4g32650 (KC1), At3G11820 (SYP121), At5G61210 (SNAP33), At1g04750 (VAMP721), and At1G12360 (SEC11).

## Supplemental Data

The following supplemental materials are available.

**Supplemental Figure S1.** KC1 and KAT1 K^+^ channel interaction controls for experiments with VAMP721, SNAP33 and SEC11.
**Supplemental Figure S2.** KAT1 K^+^ channel interacts with VAMP721, SNAP33, and SEC11 via a common N-terminal motif, RYxxWE, at the base of its voltage sensor domain.
**Supplemental Figure S3.** Pulldown analysis of the SNAREs and SEC11 with the KAT1^1–63^ cytosolic domain.
**Supplemental Figure S4.** The cytosolic domain of KAT1^1–63^ associates differentially with the cognate SNAREs in binary combinations and in complex.
**Supplemental Figure S5.** KAT1 gating is differentially sensitive to the cognate SNAREs singly and in binary combination, and the effects are moderated in tertiary combination with SEC11.
**Supplemental Figure S6.** The K^+^ channel binding domain alters SYP121 structure.
**Supplemental Figure S7.** SEC11 competes differentially with KAT1^1–63^ for cognate SNARE binding.
**Supplemental Figure S8.** SEC11 suppresses KAT1 activity stoichiometrically with SYP121 and its action is blocked at more negative voltages.
**Supplemental Table S1.** Primers and restriction enzymes used in cloning, protein interactions, and protein expression.

## References

[bib1] Archbold JK, Whitten AE, Hu SH, Collins BM, Martin JL (2014) SNARE-ing the structures of Sec1/Munc18 proteins. Curr Opin Struct Biol 29: 44–51 25282382 10.1016/j.sbi.2014.09.003

[bib2] Assaad FF, Huet Y, Mayer U, Jürgens G (2001) The cytokinesis gene *KEULE* encodes a Sec1 protein that binds the syntaxin Knolle. J Cell Biol 152: 531–543 11157980 10.1083/jcb.152.3.531 PMC2195996

[bib3] Baker RW, Hughson FM (2016) Chaperoning SNARE assembly and disassembly. Nat Rev Mol Cell Biol 17: 465–479 27301672 10.1038/nrm.2016.65 PMC5471617

[bib4] Bassham DC, Blatt MR (2008) SNAREs: Cogs and coordinators in signaling and development. Plant Physiol 147: 1504–1515 18678742 10.1104/pp.108.121129 PMC2492632

[bib5] Brunger AT, Weninger K, Bowen M, Chu S (2009) Single-molecule studies of the neuronal SNARE fusion machinery. Annu Rev Biochem 78: 903–928 19489736 10.1146/annurev.biochem.77.070306.103621 PMC2854664

[bib6] Burgoyne RD, Morgan A (2007) Membrane trafficking: Three steps to fusion. Curr Biol 17: R255–R258 17407758 10.1016/j.cub.2007.02.006

[bib7] Campanoni P, Blatt MR (2007) Membrane trafficking and polar growth in root hairs and pollen tubes. J Exp Bot 58: 65–74 16873451 10.1093/jxb/erl059

[bib8] Carpp LN, Ciufo LF, Shanks SG, Boyd A, Bryant NJ (2006) The Sec1p/Munc18 protein Vps45p binds its cognate SNARE proteins via two distinct modes. J Cell Biol 173: 927–936 16769821 10.1083/jcb.200512024 PMC3215948

[bib9] Chapman S, Faulkner C, Kaiserli E, Garcia-Mata C, Savenkov EI, Roberts AG, Oparka KJ, Christie JM (2008) The photoreversible fluorescent protein iLOV outperforms GFP as a reporter of plant virus infection. Proc Natl Acad Sci USA 105: 20038–20043 19060199 10.1073/pnas.0807551105 PMC2604982

[bib10] Christie JM, Reymond P, Powell GK, Bernasconi P, Raibekas AA, Liscum E, Briggs WR (1998) Arabidopsis NPH1: A flavoprotein with the properties of a photoreceptor for phototropism. Science 282: 1698–1701 9831559 10.1126/science.282.5394.1698

[bib11] Dawidowski D, Cafiso DS (2013) Allosteric control of Syntaxin 1a by Munc18-1: Characterization of the open and closed conformations of syntaxin. Biophys J 104: 1585–1594 23561535 10.1016/j.bpj.2013.02.004 PMC3617443

[bib12] Demircioglu FE, Burkhardt P, Fasshauer D (2014) The SM protein Sly1 accelerates assembly of the ER-Golgi SNARE complex. Proc Natl Acad Sci USA 111: 13828–13833 25189771 10.1073/pnas.1408254111 PMC4183299

[bib13] Dreyer I, Blatt MR (2009) What makes a gate? The ins and outs of Kv-like K^+^ channels in plants. Trends Plant Sci 14: 383–390 19540150 10.1016/j.tplants.2009.04.001

[bib14] Duby G, Hosy E, Fizames C, Alcon C, Costa A, Sentenac H, Thibaud JB (2008) AtKC1, a conditionally targeted Shaker-type subunit, regulates the activity of plant K^+^ channels. Plant J 53: 115–123 17976154 10.1111/j.1365-313X.2007.03324.x

[bib15] Eisenach C, Chen ZH, Grefen C, Blatt MR (2012) The trafficking protein SYP121 of Arabidopsis connects programmed stomatal closure and K^+^ channel activity with vegetative growth. Plant J 69: 241–251 21914010 10.1111/j.1365-313X.2011.04786.x

[bib16] Enami K, Ichikawa M, Uemura T, Kutsuna N, Hasezawa S, Nakagawa T, Nakano A, Sato MH (2009) Differential expression control and polarized distribution of plasma membrane-resident SYP1 SNAREs in *Arabidopsis thaliana*. Plant Cell Physiol 50: 280–289 19098073 10.1093/pcp/pcn197

[bib17] Fasshauer D, Eliason WK, Brünger AT, Jahn R (1998a) Identification of a minimal core of the synaptic SNARE complex sufficient for reversible assembly and disassembly. Biochemistry 37: 10354–10362 9671503 10.1021/bi980542h

[bib18] Fasshauer D, Sutton RB, Brunger AT, Jahn R (1998b) Conserved structural features of the synaptic fusion complex: SNARE proteins reclassified as Q- and R-SNAREs. Proc Natl Acad Sci USA 95: 15781–15786 9861047 10.1073/pnas.95.26.15781 PMC28121

[bib19] Fujiwara M, Uemura T, Ebine K, Nishimori Y, Ueda T, Nakano A, Sato MH, Fukao Y (2014) Interactomics of Qa-SNARE in *Arabidopsis thaliana*. Plant Cell Physiol 55: 781–789 24556609 10.1093/pcp/pcu038

[bib20] Gajdanowicz P, Garcia-Mata C, Sharma T, Gonzalez W, Morales-Navarro SE, Sharma t, Gonzalez-Nilo FD, Gutowicz J, Mueller-Roeber B, Blatt MR, (2009) Distinct roles of the last transmembrane domain in controlling Arabidopsis K^+^ channel activity. New Phytol 182: 380–391 19192193 10.1111/j.1469-8137.2008.02749.x

[bib21] Geelen D, Leyman B, Batoko H, Di Sansebastiano GP, Moore I, Blatt MR (2002) The abscisic acid-related SNARE homolog NtSyr1 contributes to secretion and growth: Evidence from competition with its cytosolic domain. Plant Cell 14: 387–406 11884682 10.1105/tpc.010328 PMC152920

[bib22] Grefen C, Chen ZH, Honsbein A, Donald N, Hills A, Blatt MR (2010) A novel motif essential for SNARE interaction with the K+ channel KC1 and channel gating in *Arabidopsis*. Plant Cell 22: 3076–3092 20884800 10.1105/tpc.110.077768 PMC2965544

[bib23] Grefen C, Karnik R, Larson E, Lefoulon C, Wang Y, Waghmare S, Zhang B, Hills A, Blatt MR (2015) A vesicle-trafficking protein commandeers Kv channel voltage sensors for voltage-dependent secretion. Nat Plants 1: 15108–15119 27250541 10.1038/nplants.2015.108

[bib24] Hashimoto-Sugimoto M, Higaki T, Yaeno T, Nagami A, Irie M, Fujimi M, Miyamoto M, Akita K, Negi J, Shirasu K, (2013) A Munc13-like protein in *Arabidopsis* mediates H^+^-ATPase translocation that is essential for stomatal responses. Nat Commun 4: 2215 23896897 10.1038/ncomms3215 PMC3731666

[bib25] He Y, Elias CL, Huang Y-C, Gao X, Leung Y-M, Kang Y, Xie H, Chaddock JA, Tsushima RG, Gaisano HY (2008) Botulinum neurotoxin A and neurotoxin E cleavage products of synaptosome-associated protein of 25 kd exhibit distinct actions on pancreatic islet β-cell K_v_2.1 channel gating. Pancreas 36: 10–17 18192874 10.1097/mpa.0b013e31812eee28

[bib26] Honsbein A, Blatt MR, Grefen C (2011) A molecular framework for coupling cellular volume and osmotic solute transport control. J Exp Bot 62: 2363–2370 21115662 10.1093/jxb/erq386

[bib27] Honsbein A, Sokolovski S, Grefen C, Campanoni P, Pratelli R, Paneque M, Chen ZH, Johansson I, Blatt MR (2009) A tripartite SNARE-K+ channel complex mediates in channel-dependent K+ nutrition in *Arabidopsis*. Plant Cell 21: 2859–2877 19794113 10.1105/tpc.109.066118 PMC2768940

[bib28] Horaruang W, Zhang B (2017) Mating based split-ubiquitin assay for detection of protein interactions. Bio Protoc 7: 2258 10.21769/BioProtoc.2258 PMC8410247 34541245

[bib29] Hoshi T (1995) Regulation of voltage dependence of the KAT1 channel by intracellular factors. J Gen Physiol 105: 309–328 7769379 10.1085/jgp.105.3.309 PMC2216946

[bib30] Jahn R, Scheller RH (2006) SNAREs—Engines for membrane fusion. Nat Rev Mol Cell Biol 7: 631–643 16912714 10.1038/nrm2002

[bib31] Jezek M, Blatt MR (2017) The membrane transport system of the guard cell and its integration for stomatal dynamics. Plant Physiol 174: 487–519 28408539 10.1104/pp.16.01949 PMC5462021

[bib32] Kargul J, Gansel X, Tyrrell M, Sticher L, Blatt MR (2001) Protein-binding partners of the tobacco syntaxin NtSyr1. FEBS Lett 508: 253–258 11718726 10.1016/s0014-5793(01)03089-7

[bib33] Karnik A, Karnik R, Grefen C (2013a) SDM-Assist software to design site-directed mutagenesis primers introducing “silent” restriction sites. BMC Bioinformatics 14: 105 23522286 10.1186/1471-2105-14-105 PMC3644487

[bib34] Karnik R, Grefen C, Bayne R, Honsbein A, Köhler T, Kioumourtzoglou D, Williams M, Bryant NJ, Blatt MR (2013b) Arabidopsis Sec1/Munc18 protein SEC11 is a competitive and dynamic modulator of SNARE binding and SYP121-dependent vesicle traffic. Plant Cell 25: 1368–1382 23572542 10.1105/tpc.112.108506 PMC3663274

[bib35] Karnik R, Waghmare S, Zhang B, Larson E, Lefoulon C, Gonzalez W, Blatt MR (2017) Commandeering channel voltage sensors for secretion, cell turgor, and volume control. Trends Plant Sci 22: 81–95 27818003 10.1016/j.tplants.2016.10.006 PMC5224186

[bib36] Karnik R, Zhang B, Waghmare S, Aderhold C, Grefen C, Blatt MR (2015) Binding of SEC11 indicates its role in SNARE recycling after vesicle fusion and identifies two pathways for vesicular traffic to the plasma membrane. Plant Cell 27: 675–694 25747882 10.1105/tpc.114.134429 PMC4558655

[bib37] Kwon C, Neu C, Pajonk S, Yun HS, Lipka U, Humphry M, Bau S, Straus M, Kwaaitaal M, Rampelt H, (2008) Co-option of a default secretory pathway for plant immune responses. Nature 451: 835–840 18273019 10.1038/nature06545

[bib38] Lefoulon C, Karnik R, Honsbein A, Gutla PV, Grefen C, Riedelsberger J, Poblete T, Dreyer I, Gonzalez W, Blatt MR (2014) Voltage-sensor transitions of the inward-rectifying K+ channel KAT1 indicate a latching mechanism biased by hydration within the voltage sensor. Plant Physiol 166: 960–975 25185120 10.1104/pp.114.244319 PMC4213121

[bib39] Lefoulon C, Waghmare S, Karnik R, Blatt MR (2018) Gating control and K^+^ uptake by the KAT1 K^+^ channel leaveraged through membrane anchoring of the trafficking protein SYP121. Plant Cell Environ 41: 2668–2677 29940699 10.1111/pce.13392 PMC6220998

[bib40] Lerman JC, Robblee J, Fairman R, Hughson FM (2000) Structural analysis of the neuronal SNARE protein Syntaxin-1A. Biochemistry 39: 8470–8479 10913252 10.1021/bi0003994

[bib41] Leung YM, Kwan EP, Ng B, Kang Y, Gaisano HY (2007) SNAREing voltage-gated K^+^ and ATP-sensitive K^+^ channels: Tuning β-cell excitability with Syntaxin-1A and other exocytotic proteins. Endocr Rev 28: 653–663 17878408 10.1210/er.2007-0010

[bib42] Leyman B, Geelen D, Blatt MR (2000) Localization and control of expression of Nt-Syr1, a tobacco SNARE protein. Plant J 24: 369–381 11069710 10.1046/j.1365-313x.2000.00886.x

[bib43] Leyman B, Geelen D, Quintero FJ, Blatt MR (1999) A tobacco syntaxin with a role in hormonal control of guard cell ion channels. Science 283: 537–540 9915701 10.1126/science.283.5401.537

[bib44] Lipka V, Kwon C, Panstruga R (2007) SNARE-ware: The role of SNARE-domain proteins in plant biology. Annu Rev Cell Dev Biol 23: 147–174 17506694 10.1146/annurev.cellbio.23.090506.123529

[bib45] Ma C, Su L, Seven AB, Xu Y, Rizo J (2013) Reconstitution of the vital functions of Munc18 and Munc13 in neurotransmitter release. Science 339: 421–425 23258414 10.1126/science.1230473 PMC3733786

[bib46] MacDonald PE, Wang G, Tsuk S, Dodo C, Kang Y, Tang L, Wheeler MB, Cattral MS, Lakey JRT, Salapatek AMF, (2002) Synaptosome-associated protein of 25 kilodaltons modulates Kv2.1 voltage-dependent K^+^ channels in neuroendocrine islet β-cells through an interaction with the channel N terminus. Mol Endocrinol 16: 2452–2461 12403834 10.1210/me.2002-0058

[bib47] Misura KMS, Scheller RH, Weis WI (2001) Self-association of the H3 region of Syntaxin 1A. Implications for intermediates in SNARE complex assembly. J Biol Chem 276: 13273–13282 11118447 10.1074/jbc.M009636200

[bib48] Palovcak E, Delemotte L, Klein ML, Carnevale V (2014) Evolutionary imprint of activation: The design principles of VSDs. J Gen Physiol 143: 145–156 24470486 10.1085/jgp.201311103 PMC4001776

[bib49] Pratelli R, Sutter JU, Blatt MR (2004) A new catch in the SNARE. Trends Plant Sci 9: 187–195 15063869 10.1016/j.tplants.2004.02.007

[bib50] Rizo J, Südhof TC (2012) The membrane fusion enigma: SNAREs, Sec1/Munc18 proteins, and their accomplices—Guilty as charged? Annu Rev Cell Dev Biol 28: 279–308 23057743 10.1146/annurev-cellbio-101011-155818

[bib51] Sanderfoot A (2007) Increases in the number of SNARE genes parallels the rise of multicellularity among the green plants. Plant Physiol 144: 6–17 17369437 10.1104/pp.106.092973 PMC1913785

[bib52] Shope JC, Mott KA (2006) Membrane trafficking and osmotically induced volume changes in guard cells. J Exp Bot 57: 4123–4131 17088361 10.1093/jxb/erl187

[bib53] Sieber JJ, Willig KI, Heintzmann R, Hell SW, Lang T (2006) The SNARE motif is essential for the formation of syntaxin clusters in the plasma membrane. Biophys J 90: 2843–2851 16443657 10.1529/biophysj.105.079574 PMC1414554

[bib54] Sieber JJ, Willig KI, Kutzner C, Gerding-Reimers C, Harke B, Donnert G, Rammner B, Eggeling C, Hell SW, Grubmüller H, (2007) Anatomy and dynamics of a supramolecular membrane protein cluster. Science 317: 1072–1076 17717182 10.1126/science.1141727

[bib55] Südhof TC, Rothman JE (2009) Membrane fusion: Grappling with SNARE and SM proteins. Science 323: 474–477 19164740 10.1126/science.1161748 PMC3736821

[bib56] Tombola F, Pathak MM, Isacoff EY (2006) How does voltage open an ion channel? Annu Rev Cell Dev Biol 22: 23–52 16704338 10.1146/annurev.cellbio.21.020404.145837

[bib57] Tsuk S, Lvov A, Michaelevski I, Chikvashvili D, Lotan I (2008) Formation of the full SNARE complex eliminates interactions of its individual protein components with the Kv2.1 channel. Biochemistry 47: 8342–8349 18636750 10.1021/bi800512p

[bib58] Tyrrell M, Campanoni P, Sutter JU, Pratelli R, Paneque M, Sokolovski S, Blatt MR (2007) Selective targeting of plasma membrane and tonoplast traffic by inhibitory (dominant-negative) SNARE fragments. Plant J 51: 1099–1115 17662029 10.1111/j.1365-313X.2007.03206.x

[bib59] Véry AA, Sentenac H (2003) Molecular mechanisms and regulation of K^+^ transport in higher plants. Annu Rev Plant Biol 54: 575–603 14503004 10.1146/annurev.arplant.54.031902.134831

[bib60] Wang S, Li Y, Gong J, Ye S, Yang X, Zhang R, Ma C (2019) Munc18 and Munc13 serve as a functional template to orchestrate neuronal SNARE complex assembly. Nat Commun 10: 69 30622273 10.1038/s41467-018-08028-6 PMC6325239

[bib61] Weber T, Zemelman BV, McNew JA, Westermann B, Gmachl M, Parlati F, Söllner TH, Rothman JE (1998) SNAREpins: Minimal machinery for membrane fusion. Cell 92: 759–772 9529252 10.1016/s0092-8674(00)81404-x

[bib62] Weiss N, Zamponi GW (2012) Regulation of voltage-gated calcium channels by synaptic proteins. In MS Islam, ed, Calcium Signaling, Advances in Experimental Medicine and Biology, Vol 740. Springer, New York, pp 759–775 10.1007/978-94-007-2888-2_33 22453968

[bib63] Xu J, Li HD, Chen LQ, Wang Y, Liu LL, He L, Wu WH (2006) A protein kinase, interacting with two calcineurin B-like proteins, regulates K^+^ transporter AKT1 in *Arabidopsis*. Cell 125: 1347–1360 16814720 10.1016/j.cell.2006.06.011

[bib64] Zhang B, Karnik R, Alvim J, Donald N, Blatt MR (2019) Dual sites for SEC11 on the SNARE SYP121 implicate a binding exchange during secretory traffic. Plant Physiol 180: 228–239 30850468 10.1104/pp.18.01315 PMC6501095

[bib65] Zhang B, Karnik R, Donald N, Blatt MR (2018) A GPI signal peptide-anchored split-ubiquitin (GPS) system for detecting soluble bait protein interactions at the membrane. Plant Physiol 178: 13–17 30037807 10.1104/pp.18.00577 PMC6130019

[bib66] Zhang B, Karnik R, Waghmare S, Donald N, Blatt MR (2017) VAMP721 conformations unmask an extended motif for K^+^ channel binding and gating control. Plant Physiol 173: 536–551 27821719 10.1104/pp.16.01549 PMC5210753

[bib67] Zhang B, Karnik R, Wang Y, Wallmeroth N, Blatt MR, Grefen C (2015) The Arabidopsis R-SNARE VAMP721 interacts with KAT1 and KC1 K^+^ channels to moderate K^+^ current at the plasma membrane. Plant Cell 27: 1697–1717 26002867 10.1105/tpc.15.00305 PMC4498211

